# Restricted T-Cell Repertoire in the Epicardial Adipose Tissue of Non-ST Segment Elevation Myocardial Infarction Patients

**DOI:** 10.3389/fimmu.2022.845526

**Published:** 2022-07-08

**Authors:** Daniela Pedicino, Anna Severino, Gabriele Di Sante, Maria Cristina De Rosa, Davide Pirolli, Ramona Vinci, Vincenzo Pazzano, Ada F. Giglio, Francesco Trotta, Giulio Russo, Aureliano Ruggio, Eugenia Pisano, Alessia d’Aiello, Francesco Canonico, Pellegrino Ciampi, Domenico Cianflone, Lorenzo Cianfanelli, Maria Chiara Grimaldi, Simone Filomia, Nicola Luciani, Franco Glieca, Piergiorgio Bruno, Massimo Massetti, Francesco Ria, Filippo Crea, Giovanna Liuzzo

**Affiliations:** ^1^ Dipartimento di Scienze Cardiovascolari, Fondazione Policlinico Universitario A. Gemelli Istituto di Ricovero e Cura a Carattere Scientifico (IRCCS), Rome, Italy; ^2^ Dipartimento di Scienze Cardiovascolari e Pneumologiche, Università Cattolica del Sacro Cuore, Rome, Italy; ^3^ Dipartimento di Medicina e Chirurgia traslazionale, Università Cattolica del Sacro Cuore, Rome, Italy; ^4^ Dipartimento di Medicina e Chirurgia, Sezione di Anatomia Umana, Clinica e Forense, Università di Perugia, Perugia, Italy; ^5^ Istituto di Scienze e Tecnologie Chimiche “Giulio Natta” (SCITEC) - Consiglio Nazionale delle Ricerche (CNR), Rome, Italy; ^6^ Paediatric Cardiology and Cardiac Arrhythmia/Syncope Unit, Bambino Gesù Children’s Hospital Istituto di Ricovero e Cura a Carattere Scientifico (IRCCS), Rome, Italy; ^7^ Dipartimento di Cardiologia, Aziende Socio Sanitarie Territoriali (ASST) Fatebenefratelli Sacco, Milano, Italy; ^8^ Cardiology Unit “F. Perinei” Hospital, Bari, Italy; ^9^ Cardiac Rehabilitation Unit, Istituto di Ricovero e Cura a Carattere Scientifico (IRCCS) Ospedale San Raffaele, Università Vita-Salute San Raffaele, Milan, Italy; ^10^ Dipartimento di Scienze di Laboratorio ed Infettivologiche, Fondazione Policlinico Universitario A. Gemelli Istituto di Ricovero e Cura a Carattere Scientifico (IRCCS), Rome, Italy

**Keywords:** epicardial adipose tissue (EAT), NSTE ACS, T-cell receptor (TCR), immune response, precision medicine, first acute myocardial infarction, antigen-driven immunity, computational modeling

## Abstract

**Aims:**

Human epicardial adipose tissue, a dynamic source of multiple bioactive factors, holds a close functional and anatomic relationship with the epicardial coronary arteries and communicates with the coronary artery wall through paracrine and vasocrine secretions. We explored the hypothesis that T-cell recruitment into epicardial adipose tissue (EAT) in patients with non-ST segment elevation myocardial infarction (NSTEMI) could be part of a specific antigen-driven response implicated in acute coronary syndrome onset and progression.

**Methods and Results:**

We enrolled 32 NSTEMI patients and 34 chronic coronary syndrome (CCS) patients undergoing coronary artery bypass grafting (CABG) and 12 mitral valve disease (MVD) patients undergoing surgery. We performed EAT proteome profiling on pooled specimens from three NSTEMI and three CCS patients. We performed T-cell receptor (TCR) spectratyping and CDR3 sequencing in EAT and peripheral blood mononuclear cells of 29 NSTEMI, 31 CCS, and 12 MVD patients. We then used computational modeling studies to predict interactions of the TCR beta chain variable region (TRBV) and explore sequence alignments. The EAT proteome profiling displayed a higher content of pro-inflammatory molecules (CD31, CHI3L1, CRP, EMPRINN, ENG, IL-17, IL-33, MMP-9, MPO, NGAL, RBP-4, RETN, VDB) in NSTEMI as compared to CCS (*P* < 0.0001). CDR3-beta spectratyping showed a TRBV21 enrichment in EAT of NSTEMI (12/29 patients; 41%) as compared with CCS (1/31 patients; 3%) and MVD (none) (ANOVA for trend *P* < 0.001). Of note, 11/12 (92%) NSTEMI patients with TRBV21 perturbation were at their first manifestation of ACS. Four patients with the first event shared a distinctive TRBV21-CDR3 sequence of 178 bp length and 2/4 were carriers of the human leukocyte antigen (HLA)-A*03:01 allele. A 3D analysis predicted the most likely epitope able to bind HLA-A3*01 and interact with the TRBV21-CDR3 sequence of 178 bp length, while the alignment results were consistent with microbial DNA sequences.

**Conclusions:**

Our study revealed a unique immune signature of the epicardial adipose tissue, which led to a 3D modeling of the TCRBV/peptide/HLA-A3 complex, in acute coronary syndrome patients at their first event, paving the way for epitope-driven therapeutic strategies.

## Introduction

Human epicardial adipose tissue (EAT), a dynamic source of multiple bioactive factors, owns a close functional and anatomic relationship with the epicardial coronary arteries and communicates with the coronary artery wall through paracrine and vasocrine secretions (1). Several studies suggesting the role of EAT in the pathogenesis of atherosclerosis consistently reported that EAT thickness is an independent indicator of cardiovascular risk (2, 3).

Cytokine release and pro-inflammatory cell infiltration of macrophages, lymphocytes, and basophils have been associated with the EAT of patients with established coronary artery disease who underwent elective coronary artery bypass graft (CABG) (4, 5); in these patients, macrophage polarization in EAT is shifted toward the pro-inflammatory M1 phenotype (6, 7).

Although less abundant than macrophages, T cells orchestrate the antigen-specific immune response in the coronary plaque (8–10). This occurs after the T-cell receptor (TCR) has mediated the recognition of short peptides on the human leukocyte antigen (HLA) on presenting cells. The enormous TCR diversity allows for the recognition of a wide range of potential pathogenic molecules and accounts for the difficulties in determining the antigen specificity of each receptor (11, 12).

In the present study, we sought to investigate if T-cell recruitment within EAT in acute coronary syndrome (ACS) patients with non-ST segment elevation myocardial infarction (NSTEMI) might be part of a specific antigen-driven response potentially implicated in ACS onset and progression. To this aim, we performed the EAT proteome profiling and an extended analysis of the TCR beta chain variable region (TRBV) in EAT using the T-cell repertoire of peripheral blood mononuclear cells (PBMCs) as a reference for selective EAT enrichment which led to the design of a 3D model of the cognate/specific peptide-major histocompatibility complex (MHC) target, associated with the first acute coronary event.

## Methods

For a detailed description of the methods, see the **Online Supplementary Material**.

### Study Population Design

**Figure 1:**
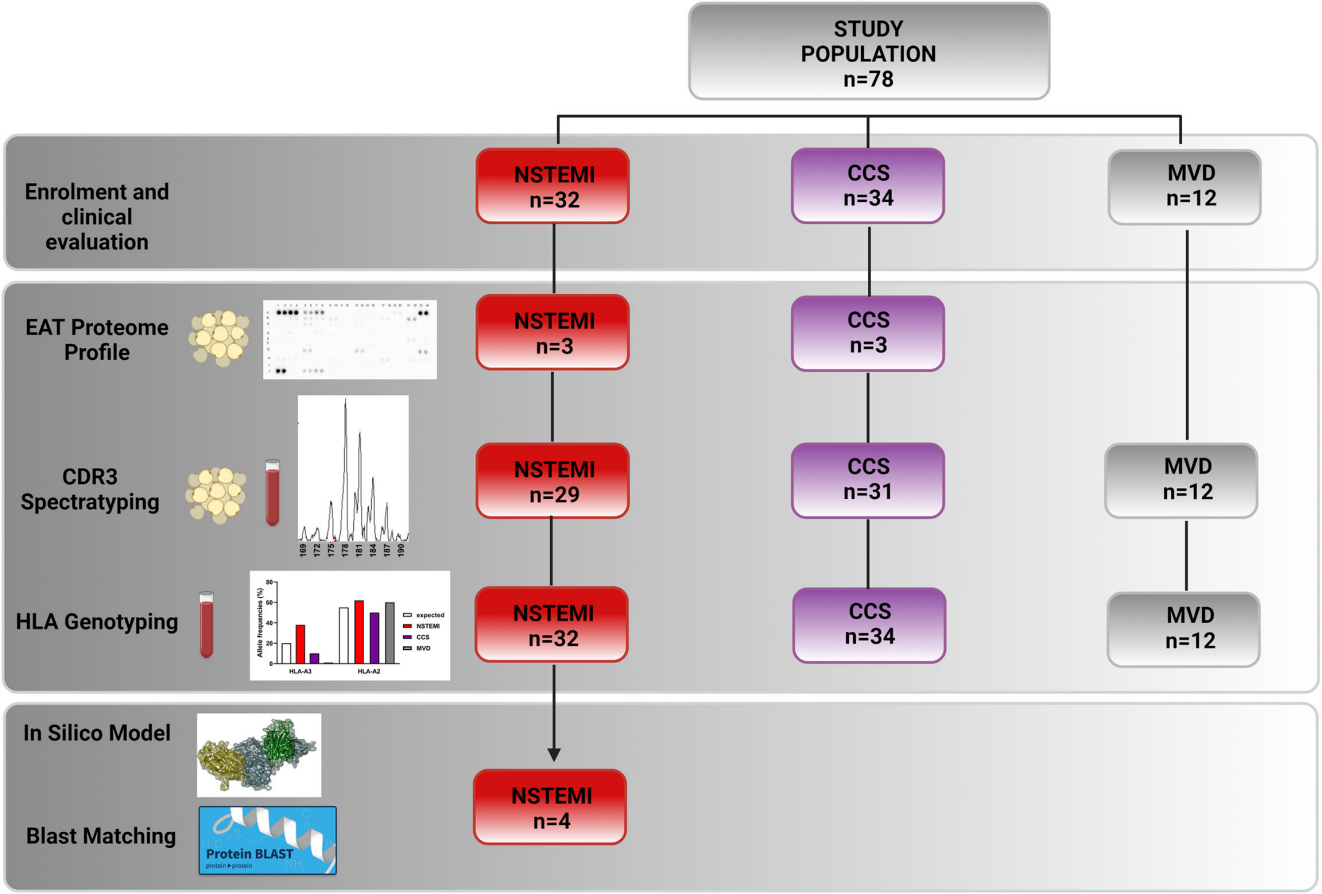
Flowchart showing enrolled patients and their experimental and study result allocations. We obtained EAT biopsies and peripheral blood from 32 NSTEMI patients, 34 CCS patients, and 12 MVD patients undergoing surgery. Three EAT specimens from NSTEMI and CCS patients were used for proteome profiling. We therefore performed TCR spectratyping and CDR3 sequencing in EAT and PBMCs of 29 NSTEMI, 31 CCS, and 12 MVD patients. We performed HLA-A molecular typing from DNA extracted from the PBMCs of the same patients. Twelve NSTEMI patients showed a TRBV21 perturbation, four of which shared a distinctive TRBV21-CDR3 sequence of 178 bp length and 2/4 were carriers of the human leukocyte antigen (HLA)-A*03:01 allele. We then used computational 3D modeling studies to predict interactions between HLA-A3*01 and the TRBV21-CDR3 sequence of 178 bp length, employing BLAST to compare the predicted peptide sequence to specific microbial databases. BLAST, Basic Local Alignment Search Tool; EAT, epicardial adipose tissue; HLA, human leukocyte antigen; MVD, mitral valve disease; NSTEMI, non-ST elevation myocardial infarction; PBMC, peripheral blood mononuclear cells; CCS, chronic coronary syndrome; TCR, T-cell receptor.

We enrolled 1) 32 patients admitted to our Coronary Care Unit with a diagnosis of NSTEMI, who underwent CABG within 14 days of symptom onset, either at their first manifestation (*n* = 19) or with previous acute coronary events (*n* = 13); 2) 34 patients with a history of chronic stable effort angina (CCS) lasting more than 12 months, severe coronary artery disease (CAD) requiring CABG, and no clinically evident effort or rest ischemic episodes during the previous 2 weeks; and 3) 12 patients presenting with mitral valve disease (MVD) undergoing cardiac surgery for mitral valve regurgitation due to degenerative disease, with angiographically normal coronary arteries. **Figure 1** displays a schematic allocation of the study population in each experimental setting. For a detailed description of the inclusion and exclusion criteria, see the **Online Supplementary Material**. The Clinical and Research Ethics Committee of Fondazione Policlinico A. Gemelli-IRCCS and the Catholic University of the Sacred Heart of Rome approved the study protocol (protocol no. 2047) that has been conducted in accordance with the principles of the Declaration of Helsinki. All participants provided their written informed consent.

### Peripheral Blood Sampling

Venous blood samples were taken a day before the surgery. PBMCs were obtained from whole blood by density gradient centrifugation over Ficoll-Hypaque (GE Healthcare Bio-Sciences, Piscataway, NJ, USA) and stored at −80°C for RNA extraction. Coded serum samples were stored at −80°C and analyzed for high-sensitivity C-reactive protein (hs-CRP) in a single batch at the end of the study. In NSTEMI patients, serum cardiac troponin I (cTnI) was determined at the time of hospital admission as a routine measurement. All categorization and management of patients were independent from these results.

### EAT Sampling and Profiling

EAT biopsy (average 0.9 g) was collected before surgery in all cases. In CCS and MVD patients, EAT was collected near the proximal right coronary artery, while in NSTEMI patients, it was collected near the culprit coronary artery, i.e., left coronary artery (LCA) in 20 cases and right coronary artery (RCA) in 12 cases. Liquid nitrogen EAT specimens were stored at −80°C and subsequently used for RNA isolation. Isolated and stimulated EAT T cells were stained with fluorochrome-conjugated mAbs anti-CD4-FITC and anti-CD8-PE-Cy5 (all from Beckman Coulter, Brea, CA, USA). A total of 100,000 events have been acquired. Non-specific staining with isotype-matched control mAb was <1%; the intra- and interassay variability was <10%. Flow cytometry analysis was conducted with FC 500 (Beckman Coulter, Brea, CA, USA) and the data were analyzed with Kaluza software (Beckman Coulter, Brea, CA, USA). EAT biopsies (average weight = 0.2 g) from CCS (*n* = 3) and NSTEMI (*n* = 3), collected as described above, were pooled to evaluate the inflammatory proteome profile (Proteome Profiler Array, R&D, USA). Details of EAT T-cell isolation and stimulation and profiling are described in the **Online Supplementary Material**.

### TCR Repertoire Analysis and CDR3 Sequencing

TCR repertoire analysis and CDR3 sequencing were performed as previously published (13–15). The method for quantifying TCR repertoire perturbation in EAT as compared with PBMCs was adapted from Gorochov et al. (16) (see the **Online Supplementary Material** and **Figure S1**).

### DNA Extraction and HLA-A Genotyping

Genomic DNA from peripheral whole blood was extracted by QIAamp DNA Mini kits (Qiagen GmbH, Hilden, Germany) and 0.1 μg of purified genomic DNA was used for HLA-DRB1 exon PCR amplification. After PCR amplification, HLA-A molecular typing was performed by a reverse hybridization method using the INNO-LiPA HLA-A kit (Fujirebio, Tokyo, Japan), following the manufacturer’s instructions. Interpretation of hybridization of HLA-A probes was made using the LiRAS software (Fujirebio, Tokyo, Japan) to predict one-digit HLA.

### *In-Silico* 3D Modeling of the TRBV/Peptide/HLA-A3 Complex

We employed a computational modeling study to predict TRBV/peptide/MHC interactions (17, 18). The homology modeling algorithm MODELLER v9.10 as implemented in Discovery Studio 4.0 (Dassault Systèmes, San Diego, CA, USA) was used to generate the computational model structure of TRBV21* (19). The 3D structure of the human anti-pre-pro insulin (PPI) protein T-cell receptor (1E6) bound to an HLA-A*0201-restricted glucose-sensitive PPI peptide (PDB code: 3UTT REF 10.1038/ni.2206), showing 70.0% sequence identity, was used as template (**Online Supplementary Material Figure S2**). The best-ranked model based on the probability density function (PDF) was selected, and the quality of the structure was assessed by PROCHECK and VERIFY3D (**Online Supplementary Material Figures S3**,**S4**). The structure of the human MHC class I molecule HLA-A*0301 (HLA-A3), in complex with a peptide (KLIETYFSK) from proteolipid protein (PDB code: 2XPG REF 10.1107/S0907444911007888), was used as the interaction partner for the modeled TRBV21*. The quaternary complex 1BD2 was used as a reference for the relative orientation of the interacting structures (20). Following the replacement of each residue by glycine (glycine), the peptide backbone was used to build putative epitope peptides by side chain construction and CHARMM’s energy minimization. This process was automatically performed by the Grow Scaffold module in Discovery Studio 4.0 by identifying the top-ranking residue in each position. After calculating and scoring, the best peptide to act as a ligand was selected for further analyses (see the **Online Supplementary Material** for references).

### Sequence Alignment and Similarity Analysis

The Basic Local Alignment Search Tool (BLAST) (21) was used to compare the predicted peptide sequence to specific microbial databases and to calculate the statistical significance of matches (see the **Online Supplementary Material**).

### Statistical Analysis

Categorical variables were described as numbers and percentages (%), and they were analyzed using the chi-square (*χ* ^2^) test or Fisher’s test, depending on sample size restrictions. The continuous variables that were normally distributed, as assessed by the Shapiro–Wilk test, were described as mean ± SD and analyzed with parametric tests. For comparisons among the three groups, we used one-way (or two-way) analysis of variance (ANOVA) with Bonferroni or Sidak correction. For between-group comparisons, we used unpaired or paired Student’s *t*-test. Data that did not follow a normal distribution were described as median and interquartile range and analyzed by using a non-parametric test. We used the Kruskal–Wallis non-parametric ANOVA and the Dunn’s test for comparisons among groups. For between-group comparisons, we used the Mann–Whitney *U*-test. To compare two related samples within groups, we used the Wilcoxon test. A two-tailed *P*-value <0.05 was considered statistically significant. Statistical analysis was performed by using SPSS Statistics 20.0 (IBM Corp., Armonk, NY, USA) and Prism software 8.02 (GraphPad, San Diego, CA 92121, USA).

## Results

The baseline characteristics of the patients are presented in **Table S1**. The study design is described in **Figure 1**.

### EAT T-Cell Infiltration and Proteome Profiling

**Figure 2:**
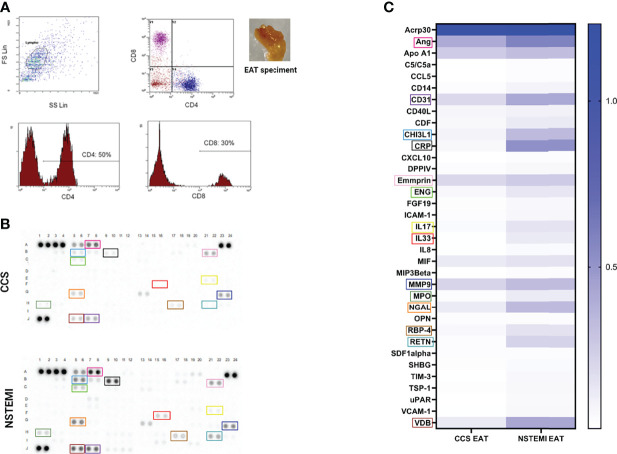
Flow cytometry characterization of EAT-resident T cells and proteome profiling of EAT specimens. **(A)** A phenotypic characterization of EAT-resident T cells was performed by flow cytometry in NSTEMI (*n* = 10) and CCS (*n* = 10) patients. A representative flow cytometry analysis is displayed and the EAT specimen of one patient is shown. About 50% of EAT-infiltrating T cells were CD4^+^, while 30% were CD8^+^ without differences among groups. **(B)** An inflammatory proteome profiling was performed in NSTEMI (*n* = 3) and CCS (*n* = 3) patients; X-ray films **(B)** and heatmap analyses **(C)** are shown [squares of the same color indicate the same molecule in **(B**, **C)**]. NSTEMI and CCS patients significantly differed for multiple immune-related molecules.

In order to characterize the presence of immune infiltrates in EAT, we analyzed the cell suspensions obtained from EAT specimens of NSTEMI (*n* = 10) and CCS (*n* = 10) patients by flow cytometry. We observed that 50% of EAT-infiltrating T cells were CD4^+^, while the 30% were CD8^+^ without differences between groups (**Figure 2A**).

To determine the relative amount of inflammatory mediators at the local level, proteome profiling of pooled EAT specimens from NSTEMI (*n* = 3) and CCS (*n* = 3) patients was performed. The results displayed highly significant differences between the two groups for multiple molecules involved in the pro-inflammatory response, cell recruitment and adhesion to the arterial wall, and vascular remodeling (**Figures 2B, C** **;** **Table S2**), thus highlighting the unique composition of the EAT in patients with NSTEMI.

### T Cells in EAT Display a Broad TCR Repertoire

**Figure 3:**
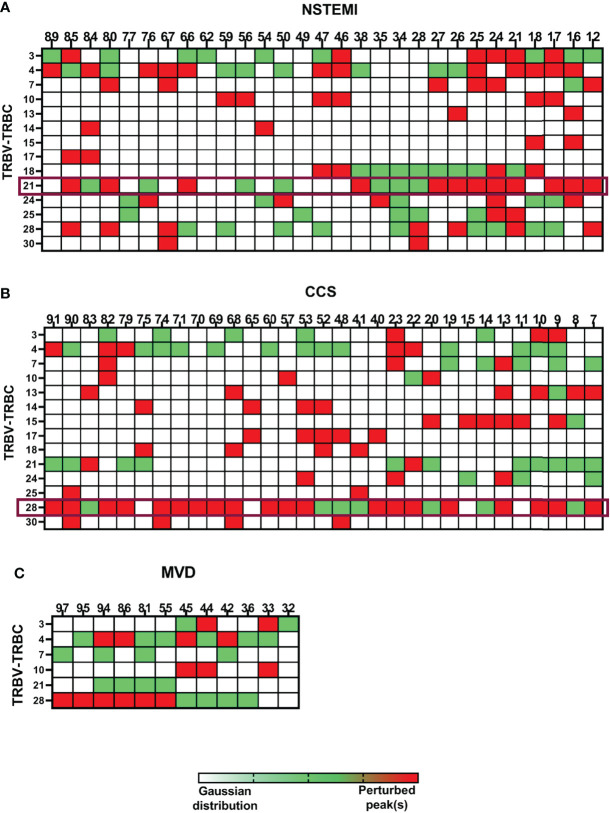
Quantitative analysis of TCR repertoire perturbations. The TRBV-TRBC spectratyping technique was applied to analyze the TCR repertoires of the enrolled patients. EAT samples were compared with the respective PBMCs. Three matrixes are displayed, one for each group of patients [**(A)** for NSTEMI, **(B)** for CCS, and **(C)** for MVD]. Single patients are displayed in columns, while the rows represent the same TRBV-TRBC TCR recombination for all patients in each group. Each square of the matrixes represents a TRBV-TRBC rearrangement for every single patient. TRBV-TRBC spectratypes from an ideal naive TCR repertoire follow an approximate Gaussian distribution containing eight or more peaks. Skewed TRBV-TRBC profiles can be detected as perturbation of this distribution. White squares represent non-perturbed Gaussian. Green squares display minimally perturbed Gaussian. Red squares indicate a high enrichment of the same peak(s) in the EAT sample compared with the homologous PBMC sample. Despite the high variability of the TCR repertoire used by each individual, it was possible to detect specific TCR signatures characterizing NSTEMI, CCS, or MVD patients.

To deepen the characterization of T lymphocyte infiltrates, a TRBV-TRBJ spectratyping was performed. We examined the size distribution of the TCR CDR3 region for 25 BV families by immunoscope spectratyping (**Table S3**). A total of 3,600 spectra were analyzed in PBMC and EAT samples from 72 patients (NSTEMI, *n* = 29; CCS, *n* = 31; MVD, *n* = 12). PBMCs and EAT T cells displayed no difference in TCR BV usage pattern. EAT obtained from MVD patients showed a comparable width of T-cell repertoire. The repertoire used by each individual is highly variable, although among the 25 BV families (**Table S3**) analyzed, 14 were used more consistently among SA and NSTEMI patients, while only 6 BV among MVD patients (**Figure 3** and **Figure S5**). Despite the high variability of the TCR repertoire used by each individual, it was possible to detect several TCR signatures characterizing specifically NSTEMI, CCS, or MVD patients.

### Perturbation of the TCR Repertoire (TRBV21) in EAT Is Associated With the First Manifestation of NSTEMI

**Figure 4:**
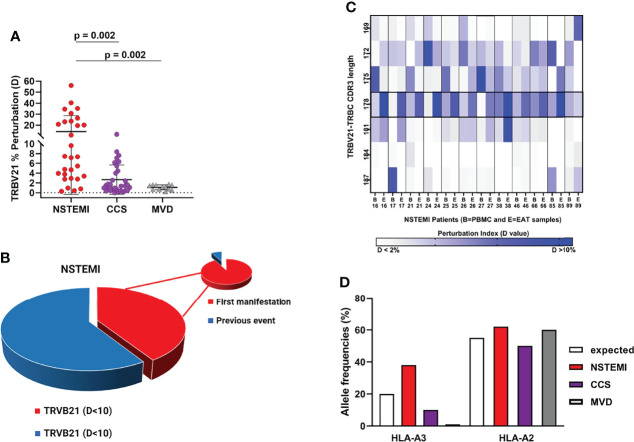
Quantitative analysis of TRBV21 perturbation. **(A)** Comparison of the TRBV21 perturbations in the three groups of patients. The TRBV21 average perturbation between EAT and PBMC samples is different in the three groups of patients (ANOVA for trends *P* < 0.001). It is significantly higher in NSTEMI patients as compared with CCS and MVD patients (*P* < 0.01 for both comparisons), but similar between CCS and MVD patients. This analysis revealed a perturbation threshold of 10% for TRBV21. **(B)** Pie chart showing the percentage of NSTEMI with TRVB21 *D >*10% (41%) and the proportion of patients at their first event (pie of pie chart) (92%). **(C)** Quantitative analysis of TRBV21 perturbation in NSTEMI patients. NSTEMI patients with high perturbation of TRBV21 (*D* > 10%) are displayed in the matrix with dark blue rectangles, highlighting that the most frequently perturbed peak is the 178-bp length. **(D)** Frequency (%) of HLA-A*02+ and HLA-A*03+ in the three groups of patients tested. HLA-A*03^+^ frequency is higher in the NSTEMI group (38%) than in the CCS group (10%). In MVD patients, A3 was not present in the analyzed cohort. The graph shows the expected frequency of HLA-A in a reference Caucasian population deduced from http://allelefrequencies.net (>110,000 individuals). As a referral, we also reported the frequency of the commonly used HLA in the Caucasian population.

Focusing on the most shared TCRs, we calculated using algorithms the threshold values and determined the cutoffs to identify specific disease-related TCRs as biomarkers. The perturbation (*D*) degree (%) of the TCR repertoire was calculated for each patient in PBMC and EAT specimens as a function of the difference between the *P* distribution of the EAT sample and the *P* distribution of the PBMC sample (reference sample). This approach provides a quantitative determination of repertoire perturbations with a *D* value carrying from 0% to 100% (**Online Supplementary Material, Figure S1**). As illustrated in **Figure 3** and **Figure S5**, the perturbations of the TCR repertoire in EAT are distributed differently in NSTEMI and CCS patients: in NSTEMI patients, alterations of TRBV21 were strongly prevalent, while perturbations of TRBV28 were observed in CCS and MVD patients, although not statistically significant. Indeed, TRBV21 perturbation was significantly higher in NSTEMI (median, range: 7.4, 0.3–56) as compared to CCS (1.4, 0.02–11.6) and MVD (1.2, 0.2–1.7; ANOVA by Kruskal–Wallis *P* < 0.001; Dunn’s multiple comparisons test: both *P* = 0.002) (**Figure 4A**). A TRBV21* perturbation (*D* > 10%) was observed in 12/29 (41%) of NSTEMI patients (compared with an expected 1%, in a random use of TCR gene segments) (22). TRBV21 was not enriched in EAT of CCS and MVD patients (3% and 0%, respectively; ANOVA for trend *P* < 0.001). Of note, 11/12 (92%) NSTEMI patients with TRBV21* perturbation (*D* > 10%) were at their first manifestation and only one patient (8%) had previous acute coronary events (*P* = 0.008) (**Figure 4B**). **Figure S6** shows the receiver-operating characteristic (ROC) curves for TRBV21* perturbation. Furthermore, most perturbations of the TRBV21 distribution in NSTEMI patients focused on one single CDR3 of 178 bp length (**Figure 4C** and **Figure S7**). The same analysis was performed for TRBV28 (**Online Supplementary Material Figure S8**), demonstrating the clonal peculiarity of EAT T cells.

### The NSTEMI-Associated TRBV21 Family Shares a Common CDR3 Sequence

To understand whether the enriched NSTEMI-associated TCR shared the same hypervariable region, several sequencing analyses were performed. Each CDR3 BV peak contains thousands of different sequences, each coding for a unique TCR with a particular antigen specificity. To determine whether the perturbation observed for EAT CDR3 profiles reflected a clonal expansion of T cells, CDR3 sequences of EAT samples were analyzed. Two hundred and eighty-eight sequences were obtained from nine NSTEMI patients, after a screening of about 50 plasmids per sample. Surprisingly, the same sequence (TRBV21 CASSKA ETDE ETQYFGPGTRL) was obtained in four out of the nine NSTEMI patients, all at their first manifestation. This observation supported the idea that T cells carrying this TCR were selectively enriched or expanded in EAT, at the onset of NSTEMI.

A similar CDR3 sequence analysis was carried out with the BV28 family for which several perturbations were also observed in CDR3 spectratyping profiles of CCS patients. In this case, however, we could not find sequences frequently recurring among samples. The resulting sequences and the expected length for TRBV21 and TRBV28 are reported in the **Online Supplementary Material Tables S4**–**S6**.

### HLA-A Genotyping

In Caucasians, the most frequent HLA alleles belong to the HLA-A2 family, which collectively has an allelic frequency of approximately 30%, leading to an ≅50% of individuals being HLA-A2 positive in the population (23).

However, two of four NSTEMI patients at their first manifestation of disease with EAT samples displaying the usage of sequence TRBV21 CASSKA ETDE ETQYFGPGTRL were HLA-A*03^+^ (**Figure 4D**). These observations led us to propose HLA-A*03 as the candidate restricting element for the public TCR (TRBV21 CASSKA ETDE ETQYFGPGTRL) in our NSTEMI patients, for further studies.

### *In-Silico* 3D Modeling

**Figure 5:**
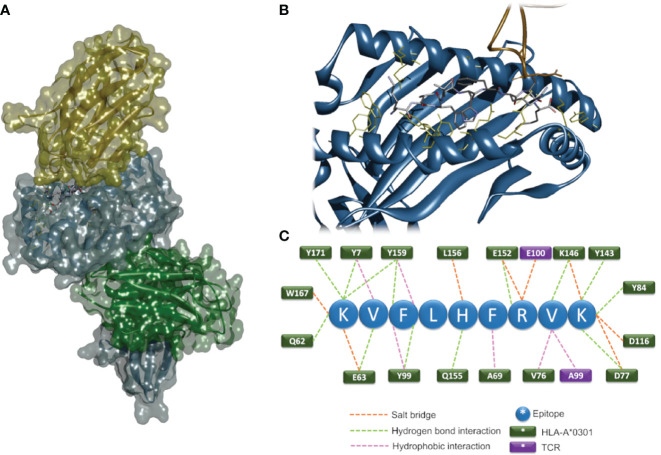
*In-silico* 3D modeling. Molecular modeling of the TCRBV/HLA-class I/epitope complex. Overall 3D structure of the quaternary complex **(A)**. The backbone structures of TCRBV21 (yellow), HLA-A3*01 α-chain (blue), and β2-microglobulin (green) are displayed in ribbon and solvent-accessible surface representations. The epitope residues are in stick representation color-coded by atom types. A zoom view of the contact interface showing the residues important for the stabilization of the complex **(B)**. Sketch of the predicted interactions at the interface **(C)**; epitope residues are shown as blue circles, and HLA and TCR residues as green and violet rectangles, respectively.

We decided to design a 3D molecular modeling strategy aimed at identifying putative antigen peptide sequence and conformation in the TCRBV/HLA-class I/epitope complex. A scoring algorithm was used to rank sequence candidates, and the top-ranking sequence KVFLHFRVK was selected as the most likely epitope able to bind HLA-A3*01 and interact with the TRBV21* CASSKA ETDE ETQYFGPGTRL. The 3D model structures of the TCRBV/HLA-class I/epitope complex and the interacting residues are shown in **Figure 5** and **Table 1**.

**Table 1:** Intermolecular non-bond interactions established by the predicted epitope with HLA-A*0301 and TCR.

Epitope	HLA-A*0301	TCR	Non-bond interactions
LYS1 (N)	TYR7 (OH)		H-bond
LYS1 (N)	TYR171 (OH)		H-bond
LYS1 (NZ)	GLN62 (OE1)		H-bond
LYS1 (NZ)	TYR159 (OH)		H-bond
LYS1 (NZ)	GLU63 (OE2)		Salt bridge
LYS1 (NZ)	TRP167 (AR)		Pi-cation
VAL2 (CG1)	TYR7		Hydrophobic
VAL2 (N)	GLU63 (OE1)		H-bond
PHE3 (AR)	TYR159 (AR)		Hydrophobic
PHE3 (AR)	TYR99 (AR)		Hydrophobic
PHE3 (N)	TYR99 (OH)		H-bond
PHE3 (OH)	TYR159 (OH)		H-bond
HIS5 (AR)	LEU156 (AR)		Hydrophobic
HIS5 (ND1)	GLN155 (OE1)		H-bond
PHE6 (AR)	ALA69 (AR)		Hydrophobic
ARG7 (NH1)	GLU152 (OE2)		Salt bridge
ARG7 (NH2)	GLU152 (OE2)		H-bond
ARG7 (NH2)	GLU100 (OE2)		Salt bridge
VAL8 (CG1)	ALA99 (CB)		Hydrophobic
VAL8 (O)	LYS146 (NZ)		H-bond
VAL8 (CG2)	VAL76 (CG1)		Hydrophobic
LYS9 (OXT)	LYS146 (NZ)		Salt bridge
LYS9 (N)	ASP77 (OD2)		Salt bridge
LYS9 (NZ)	ASP116 (OD2)		Salt bridge
LYS9 (NZ)	ASP77 (OD1)		H-bond
LYS9 (O)	THR143 (OG1)		H-bond
LYS9 (O)	TYR84 (OH)		H-bond

Intermolecular non-bond interactions established by the predicted epitope with HLA-A*0301 and TCR as identified by Discovery Studio 4.0 (Dassault Systèmes 2018). A three-letter amino acid code followed by position number is used. Atoms involved in the interaction are reported in standard PDB atom names.

### Proteins of EAT Microbiota Contain Sequences Homologous to the Putative Epitope Sequence

Our group has recently demonstrated the existence of a local microbiome signature in EAT (24). Based on this scenario, a sequence similarity analysis between the putative antigen sequence (K^1^V^2^F^3^L^4^H^5^F^6^R^7^V^8^K^9^) and the EAT microbiota genome was performed by BLASTp (21). We find that several sequences from these bacteria display homology higher than 80% with our candidate epitope sequence (**Table 2**).

**Table 2:** Sequence similarity analysis between the putative antigen sequence (K^1^V^2^F^3^L^4^H^5^F^6^R^7^V^8^K^9^) and EAT microbiota genome as performed by BLASTp (25).

*Phylum*	Genus	Accession	Protein	Peptide sequence	Max score	Total score	Query cover (%)	E-value	Identity (%)
*Actinobacteria*	*Actinomyces*	WP_075414257.1	Hypothetical protein	VFLHFR	24	24	66	4.7	100
	*Actinomyces*	BAV83756.1	Hypothetical protein	VFLHFR	24	24	66	4.7	100
	*Actinomyces*	WP_073708799.1	Oligoribonuclease	FLHFR	21	21	55	55	100
	*Propionibacterium*	WP_055345539.1	ABC transporter ATP-binding protein	IFLHFRGK	22.7	22.7	88	1.2	75
	*Propionibacterium*	SCQ71317.1	Nod factor export ATP-binding protein I (nodulation ATP-binding protein I)	IFLHFRGK	22.7	22.7	88	1.2	75
	*Propionibacterium*	SCQ60498.1	ABC daunorubicin resistance transporter, ATP-binding component (precursor)	IFLHFRGK	22.7	22.7	88	1.2	75
*Firmicutes*	*Ruminococcus*	WP_028510096.1	Hypothetical protein	LHFRVK	23.1	23.1	66	13	100
	*Ruminococcus*	CDE11995.1	cRISPR-associated protein Csd1 family	VFLRFRV	22.3	22.3	77	26	85.71
	*Ruminococcus*	WP_118609816.1	Sensor histidine kinase*	KVFLEFSVK	22.3	22.3	100	26	77.78
*Proteobacteria*	*Rickettsiales*	RPF74257.1	Glucose-1-phosphate thymidylyltransferase*	KVFLHRVK	24.8	24.8	100	1.9	88.89
	*Rickettsiales*	MAR56391.1	Hypothetical protein CMM93_04335	IFLHFR	21.8	21.8	66	22	83.33
	*Rickettsiales*	MBJ94827.1	Hypothetical protein CMP23_10205	VFMHFR	21.4	21.4	66	31	83.33
*Cyanobacteria*	*Cyanobacteria*	WP_068817593.1	RibD family protein [*Phormidesmis priestleyi*]*	VFLHYRVK	26.9	26.9	88	3.0	87.50
	*Cyanobacteria*	WP_015194555.1	RibD family protein [*Stanieria cyanosphaera*]*	VFLHYRVK	26.9	26.9	88	3.0	87.50
	*Cyanobacteria*	WP_095722341.1	RibD family protein [*Calothrix elsteri*]	VFLHYRVK	26.9	26.9	88	3.0	87.50

*Best candidate sequences as epitopes triggering the activation of T cells at thefirst episode of NSTEMI.

Among the *Firmicutes*-derived sequences, two display the RV residues in the appropriate position, with one having an R for the H^5^ in the query. Since R (arginine) and H (histidine) have distinct chemical properties, we are not certain that this peptide would be able to interact properly with the HLA-A*0301 molecule. In the sequences obtained from *Cyanobacteria* (VFLH**Y**RVK), Y substitutes for F^6^. Although this is not a conservative substitution, in our model, F^6^ interacts only with an A (alanine^69^) of HLA-A*0301, an interaction that may occur also for a Y (tyrosine) residue in the same position.

Overall, we propose that sequences from *Firmicutes*/*Ruminococcus* (LHFRVK) and *Cyanobacteria* (VFLHYRVK) display most of the characteristics required for the interaction with HLA-A*0301 and TRBV21* and can be considered good candidates as epitopes triggering the activation of T cells at the first episode of NSTEMI.

## Discussion

The close anatomical relationship between EAT and coronary arteries has always suggested a likely involvement of the adipose tissue in CAD (1). However, the functional role of the adipose tissue surrounding the heart is still barely elucidated. Several clues indicate that EAT is one of the key characteristics of CAD pathophysiology (25–27). Therefore, in addition to its storage and protective functions, EAT must be considered by all accounts a lymphatic organ, characterized by leukocyte trafficking and cytokine and adipokine release. Our NSTEMI EAT specimens effectively hold a peculiar proteome profile displaying an increased content of a bunch of pro-inflammatory molecules (CRP, IL-17, IL-33, CDF, RETN, RBP-4, CHI3L1) as well as proteins involved in cell recruitment and adhesion to the arterial wall and vascular remodeling (CD14, NGAL, CD31, MMP-9, VCAM1, MPO, ENG, ANG), thereby strengthening the EAT multifaced nature.

Indeed, the EAT of patients with CAD undergoing CABG shows high levels of pro-inflammatory cytokines and cell infiltration with lymphocytes, basophils, and macrophages (4, 5), mostly displaying the pro-inflammatory M1 phenotype (6, 7). The total amount of T lymphocytes is increased in EAT of CAD patients as compared with subcutaneous adipose tissue and EAT of non-CAD patients (28).

A sizeable proportion of patients presenting with ACS shows a unique adaptive immune system profile, characterized by higher levels of effector T cells and reduced levels and/or function of circulating T regulatory cells, together with a disproportionate TCR activation (29–31). T-cell clonal restriction has been demonstrated both in peripheral blood and in coronary thrombi of ACS patients (9, 10), suggesting a specific antigen-driven response. Given the role of adaptive immune dysregulation in the pathogenesis of ACS, EAT might likely contribute, as immunologically active tissue, to the immune unbalance leading to the unstable plaque (4).

Microbial DNA has been found in the EAT environment of ACS patients in association with the NOD-like receptor P3/inflammasome activation (24), suggesting, along with other evidence (32), that the gut-resident microbiome might directly or indirectly influence the progression toward plaque instability through an antigen-driven response. These data have brought back the “infection hypothesis,” according to which an infectious event, or even just an altered composition of gut microbiome without clinical signs of infection, could act as a trigger for ACS (33). Given this, our missing piece in the history was to understand whether and how the EAT *milieu* might contribute to the immune alterations leading to coronary plaque instability. The goal of this work was to prove that an immune response to specific antigens might occur in the EAT as one of the steps toward ACS.

T-cell accumulation in non-lymphoid tissues (EAT in our study) is shaped by several mechanisms including migration and retention of circulating T cells as well as expansion of clones specific for tissue-specific antigens. The local chemokine and cytokine milieu and the expression of specific antigens in the tissue promote chemotaxis and clonal expansion of T cells (10, 12). Here, we report for the first time that EAT of NSTEMI patients at their first clinical manifestation showed the enrichment of an exclusive TRBV21* public T-cell receptor, demonstrating a consistent pattern of clonal restriction in EAT T cells. We could also observe that the presence of TRBV21* was co-occurring with an HLA-A*03^+^ haplotype that in turn was more frequent in our NSTEMI patients when compared with CCS patients and the expected frequency in the general population. These observations reinforced the hypothesis of a specific, antigen-driven, T-cell expansion in EAT along with the first presentation of ACS.

We previously reported in human (14) and experimental (34) autoimmune diseases that the first wave of T cells specific for a given antigen during the immune response is often characterized by the use of public TCRs, leading to a skewed TCR repertoire. At later times, during chronic disease, the early T-cell repertoire can be modified by exhaustion of some of the activated antigen-specific T cells expanded by the first event (34), in tandem with epitope spreading and TCR repertoire enlargement. Thus, the memory repertoire generated following primary immunization and expanded upon secondary encounter(s) with the antigen changes its clonal composition over time, at least in part to address a presumably larger epitope repertoire. This might account for the differences observed in the TCR repertoire composition between NSTEMI patients with and without previous events.

TCRs recognize short peptides presented on the HLA. To date, several strategies have been used to determine the antigen specificities of T cells knowing the TCR sequence and the restricting element (35). In this study, we used *in-silico* molecular modeling to describe the TCRBV/HLA-class I/epitope quaternary complex and predict a putative sequence of the target epitope, starting with an unbiased interrogation of TCR specificity. Finally, this computational modeling allowed us to highlight a similarity between the putative epitope sequence and the sequences found in bacterial phyla associated with ACS and found in the gut microbiota (24). Overall, this scenario is in line with recent demonstrations of cross-reactive CD4^+^ T cells, primed by epitopes derived from microbes colonizing different mucosal tissues, able to infiltrate target organs, causing or exacerbating both autoimmune (36) and autoinflammatory diseases (37). Moreover, in agreement with our findings, another study has recently described the involvement and the activation of heart-specific Th cells by bacterial peptide mimics derived from the intestinal microbiota, able to enter the myocardium, enhancing the damage caused by infection during lethal inflammatory cardiomyopathy (38).

**Figure 6:**
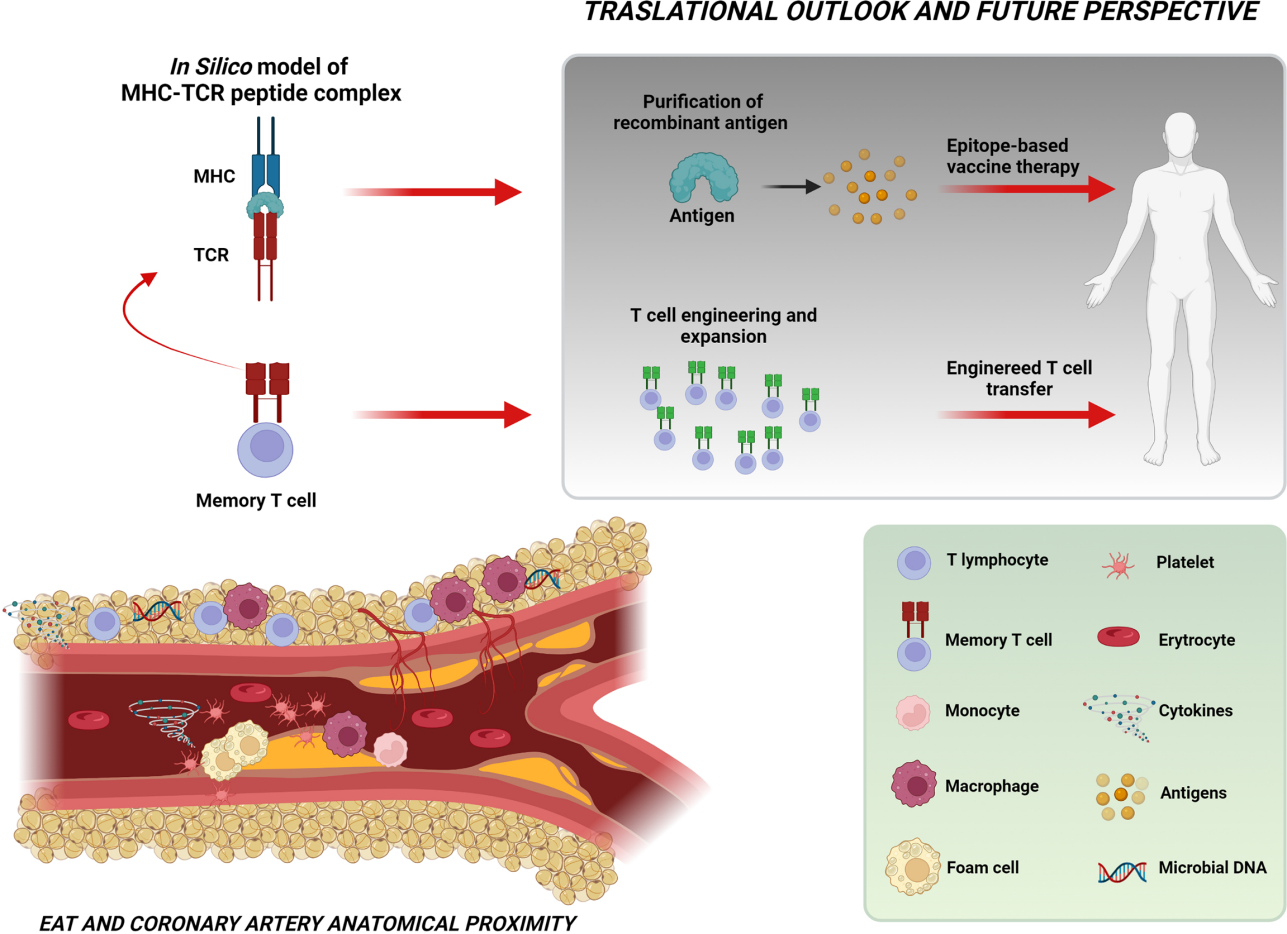
Take-home figure. The figure shows the close relationship between EAT and coronary arteries and the intricate cellular and molecular network possibly implicated in the pathogenesis of ACS. The identification of a specific T-cell clonal expansion in the EAT of NSTEMI patients at their first clinical manifestation and the prediction of the putative sequence of the MHC–TCR–peptide complex (through *in-silico* modeling) indicate the existence in the EAT of an antigen-driven immune response likely involving microbiome-derived antigens as triggers for instability. These observations represent a significant step toward the perspective of engineered T-cell or epitope-based vaccine therapies. Created with BioRender.com.

Thanks to its privileged position of close proximity to the coronary arteries, EAT represents the ideal environment for a specific T-cell clonal expansion in response to antigen exposure. Whether the enriched T cells and the antigens detected in the EAT result from direct microbial colonization or represent the consequence of previous peripheral immune responses needs to be proven in further studies. However, the evidence for an antigen-driven immune response as a molecular and cellular marker of the first coronary event represents the first step toward a personalized approach in cardiovascular medicine for the ideation of epitope-based vaccines in the treatment of ACS. In the future, more advanced methods that integrate computational biology and structural modeling might be used to design highly specific and powerful TCRs for use in T-cell therapies (**Figure 6**).

## Conclusions

The identification of a specific T-cell enrichment in the EAT of patients presenting at their first coronary event represents the clue of a specific antigen-driven immune response in the pathogenesis of ACS. Together with the available evidence on the role of dysbiosis in plaque instability, our data pave the way for the fascinating hypothesis of microbiome-derived antigens as triggers for plaque instability. These observations represent a significant step toward the intriguing perspective of engineered T-cell or epitope-based vaccine therapies that begins from a genome-based computational model and terminates with advanced, personalized healthcare (**Figure 6**).

## Limitations and Perspectives

Our study is more hypothesis-generating than hypothesis-proving by its nature. Some limitations should be recognized. First, T cells may have encountered bacteria somewhere else, and we cannot exclude that the restricted TCR diversity observed in the EAT surrounding diseased coronary arteries may reflect differential entrapment of antigen primed T cells from the circulating T-cell pool. One possibility is the cross-reactivity of T cells exposed to peptide motifs shared by the human proteome and gastrointestinal microbiota (39) or, as it happens for other autoimmune/autoinflammatory mechanisms, a TCR-independent pathway or a bystander effect (40). Second, in a previous study, we have documented the presence of bacterial DNA of gut microbiota phyla in the EAT of NSTEMI patients (24). However, we did not demonstrate a direct bacterial colonization in EAT, since bacterial DNA in EAT might represent both the clue of direct bacterial colonization and the result of antigen-presenting cell translocation following phagocytosis that occurred elsewhere. In the current study, we did not have the opportunity to directly search bacterial DNA in the EAT samples showing the related TRBV21 sequences because of the scarcity of tissue. Third, we could not directly prove that induction of pro-inflammatory signaling by the related T cells in EAT plays a role in the generation of unstable plaques. However, taking together the present work and our previous data, the demonstration of T-cell enrichment in EAT of NSTEMI patients agrees with the already demonstrated upregulation of NLRP3 inflammasome (24) and with the available evidence of an altered immune response as a trigger for plaque instability. Whether or not microbial colonization of EAT antecedes this immune response, the demonstration of enriched T cells in the EAT of NSTEMI patients at their first manifestation represents sufficient proof of an antigen-driven immune response taking place in the adipose tissue surrounding the coronary artery.

## Data Availability Statement

The datasets presented in this study can be found in online repositories. The name of the repository and link to the data can be found below: Figshare; 10.6084/m9.figshare.19932182.

## Ethics Statement

The studies involving human participants were reviewed and approved by the Clinical and Research Ethics Committee of Fondazione Policlinico A. Gemelli-IRCCS and the Catholic University of the Sacred Heart of Rome. The patients/participants provided their written informed consent to participate in this study.

## Author Contributions

GL, DPe, AS, FR and GDS designed the research. GL provided funding support. FT, AFG, Ad’A, PC, AR, and PB collected the biological materials. AS, VP, GDS, RV, FCa, and EP processed the biological materials and performed the statistical analyses. DC, LC, DPe, GR, NL, FG, PB, MCG, and SF performed the screening and selection of patients MCDR and DPi performed computational modelling. AS, DPe, FR, and GDS investigated and analyzed the data and wrote the original manuscript. DPe, AS, RV, GL, GDS, and FR reviewed and edited the final manuscript. GL, FCr, MM, and FR provided suggestions and performed critical reading of the manuscript. All authors read and approved the submitted version.

## Funding

The present study was partially supported by the Catholic University of the Sacred Heart Linea D1 2016 Grant and by the Italian National Project Grant PRIN 2017, Protocol 2017WJBKKW_001.

## Conflict of Interest

The authors declare that the research was conducted in the absence of any commercial or financial relationships that could be construed as a potential conflict of interest.

## Publisher’s Note

All claims expressed in this article are solely those of the authors and do not necessarily represent those of their affiliated organizations, or those of the publisher, the editors and the reviewers. Any product that may be evaluated in this article, or claim that may be made by its manufacturer, is not guaranteed or endorsed by the publisher.
