# Supplementary material for: Restricted T-Cell Repertoire in the Epicardial Adipose Tissue of Non-ST Segment Elevation Myocardial Infarction Patients
Source: Front Immunol. 2022 Jul 8;13:845526. doi: 10.3389/fimmu.2022.845526 (PMC9307872; doi:10.3389/fimmu.2022.845526)
Supplement: Supplementary file 1 [file DataSheet_1.docx]

Supplementary Material

**1 Supplementary Methods**

**1.1 Study population**

*Non-ST-elevation Myocardial Infarction (NSTEMI) patients.* We prospectively evaluated 33 consecutive patients admitted to our Coronary Care Unit with a diagnosis of non-ST-elevation myocardial infarction (NSTEMI), who underwent coronary artery by-pass surgery within 14 days from symptoms’ onset. Exclusion criteria were: 1) age >80 years; 2) evidence of inflammatory or infectious diseases, malignancies, immunologic or hematological disorders; 3) allergic disorders, including pulmonary asthma; 4) ejection fraction <40%; 5) treatment with anti-inflammatory drugs other than low-dose aspirin.

*Chronic coronary syndrome (CCS) patients.* During the same period, we screened 34 consecutive patients with a history of chronic stable effort angina lasting more than 12 months, severe coronary artery disease requiring coronary artery by-pass surgery, and no clinically evident ischemic episodes during the previous 2 weeks. Exclusion criteria were: 1) age >80 years; 2) evidence of inflammatory or infectious diseases, malignancies, immunologic or hematological disorders; 3) allergic disorders; 4) ejection fraction <40%; 5) treatment with anti-inflammatory drugs other than low-dose aspirin.

In all NSTEMI and CCS patients, information regarding demographic data, traditional risk factors (including smoke, diabetes mellitus, hypertension, dislipidemia), body mass index (BMI), history of previous NSTEMI, previous coronary revascularization procedures, angiographic findings, left ventricular ejection fraction, and medical treatment at the time of study enrollment were carefully recorded.

*Mitral Valve Disease (MVD) patients.* As control group, we screened 12 individuals aged >50 years undergoing cardiac surgery for mitral valve regurgitation due to degenerative disease (MVD), with angiographically normal coronary arteries. Patients with left ventricular ejection fraction < 60%, atrial fibrillation, atrial enlargement, and infective endocarditis were excluded.

Demographic and clinical characteristics of patients involved in the study are listed in Table S1. The study protocol (protocol N° 2047) was approved by the Clinical and Research Ethics Board of Fondazione Policlinico A. Gemelli-IRCCS of Rome, and the study was conducted in accordance with the principles of the Declaration of Helsinki. All participants provided written informed consent.

**1.2 Cell suspension from EAT and flow cytometry analyses**

Adipose tissue samples from NSTEMI (n = 10) and CCS (n = 10) patients were minced with sterile scissors, washed in PBS, digested by 1mg/mL% collagenase (Sigma-Aldrich, St. Louis, MO, US) for 60 min at 37 °C on a gently rocking platform and centrifuged for 12 min at 1200 × g. Finally, samples were filtered through Falcon® 40 μm Cell Strainer (Becton, Dickinson and Company, Franklin Lakes, USA) to eliminate the adipocytes. Cells were cultered in RPMI 1640 medium supplemented with 10% FCS, 1 mM sodium pyruvate, 0.1 mM non-essential amino acids, 2 mM l-glutamine, 25 mM HEPES, 100 U/ml penicillin, 100 μg/ml streptomycin (all from Lonza, Basilea, CH) in the presence of anti-CD3/CD28 coated microbeads (Invitrogen, Carlsbad, CA, USA) and IL-2 100U (Miltenyi Biotec, Bergisch Gladbach, DE) to promote T-cell expansion. After 72 hours, a total amount of 100 μl of cell suspension with average 5x10^5^ cells were stained with fluorochrome-conjugated mAbs anti CD4-FITC and anti CD8-PE-Cy5 (all from Beckman Coulter, Brea, CA, US). A total of 100,000 events were acquired. Non-specific staining with isotype-matched control mAb was <1%; the intra- and inter-assay variability was <10%. Flow-Cytometry analysis was conducted with FC 500 (Beckman Coulter, Brea, CA, US) and the data were analyzed with Kaluza software (Beckman Coulter, Brea, CA).

**1.3 EAT proteome profiling**

EAT biopsies from CCS (n = 3) and NSTEMI (n = 3) were collected before the surgery in all cases and immediately frozen at -80°C. In CCS EAT was collected near the proximal right coronary artery; in NSTEMI patients, EAT was collected near the culprit coronary artery, *i.e.* left coronary artery (LCA).

EATs were homogenized in cold radioimmunoprecipitation (RIPA) buffer (1mg/100mL) supplemented with a protease and phosphates inhibitor cocktail (1:100) (Halt^TM^ Protease and Phosphatase Inhibitor Cocktail, Thermo Fisher Scientific, USA) and by mechanical disruption (TissueLyserII, Qiagen, DE). Homogenized samples were centrifuged at 14000x g/10’/4°C and EAT protein concentration were quantified by Bradford method.

EAT samples were pooled for pro-inflammatory proteome profiling evaluation. A total of 200 micrograms per membrane of pooled EAT (CCS vs NSTEMI) were incubated. The proteome profile assay was performed according to manual user (ARY022B, Proteome Profiler Array, R&D, USA). After X-ray acquisition (ChemiDoc™, Biorad, USA), obtained spots were analyzed using Image Lab software (Biorad, USA).

**1.4 RNA extraction and reverse-transcription reaction**

Total RNA was extracted from peripheral blood mononuclear cell (PBMC) and EAT samples with RNeasy Minikit and RNeasy Lipid Tissue Mini kit respectively (Qiagen NV, Venlo, the Netherlands) according to the manufacturer’s instructions. For each sample, 1 μg of RNA was reverse-transcribed to cDNA using the iScript^TM^ cDNA Synthesys kit (Biorad, Hercules, CA, US) according to the manufacturer’s instructions.

**1.5 CDR3 Spectratyping**

Immunoscope was used to examine the size distribution of T-cell receptor (TCR) CDR3 BV chain rearrangement in 25 BV families. cDNA was amplified with each of 25 TRBV family-specific primers together with a TRBC1α primer deduced from IMGT database (ImMunoGeneTics, www.imgt.com) (Table S2). As previously published by our group^1-3^ and in line with the current literature, we used the Gene notation to indicate each BV chain product. The BV families analysed were chosen arbitrarily to cover more than 50% of the total TCR repertoire. The achieved amplicons were then used in a run-off reaction with a nested fluorescent TRBC-specific primer in order to label PCR products. For the precise determination of fragment sizes, 2 μL of the run-off products were then denatured in formamide and separated on an automated ABI PRISM 3130 DNA analyser (Thermo Fisher, ‎Waltham, MA‎, U.S). The size and intensity of each band were analysed by Gene-mapper version 4.0 software (Thermo Fisher, ‎Waltham, MA‎, U.S). Briefly, the software program identified each histogram peak by its PCR-size length and determined the area under each peak. Fluorescence intensity was plotted in arbitrary units on the y-axis, with the x-axis corresponding to CDR3β length determined in base pair (Figure S1).

**1.6 TCR perturbation Index**

Spectratypes of the CDR3 region from an ideal naive repertoire follow an approximate Gaussian distribution containing eight or more peaks. Skewed CDR3 BV profiles can be detected as perturbation of this distribution. Accordingly, spectratyping data analysis was performed with the Gene Mapper 4.0 software package (Thermo Fisher, ‎Waltham, MA‎, U.S). The method for quantifying T cell repertoire perturbation was adapted from *Gorochov et al*.^4^. Briefly, CDR3 length profiles were translated into a p distribution *p^k^(i)* for each peak *i* (*i*=1, 2,.. n**)** of a given BV**_k_** as a function of the area under the curve (expressed in relative fluorescence intensity, RFI) with normalization so that Σ*_i_p^k^(i)=*100%. D^k^(*i*) represents the extent of the perturbation for each peak(*i*) and it has been calculated the distance D^k^(*i*) between p^k^(*i*) values from the sample and p_ref_^k^(*i*) values from a reference distribution derived from repertoire analysis of an ideal Gaussian represented by the mean values for each PBMC of all the enrolled patients. The perturbation *D^k^* for a given BV_k_ was then calculated as Σ_i_| D^k^(i) |/2, so that D^k^ = 0 or 100% for a p distribution equal to or completely non overlapping with the reference, respectively (Figure S1).

**1.7 CDR3 region sequencing**

Total BV21/BC-amplified products from different cDNA were cloned by TOPO TA Cloning® kit (Thermo Fisher, ‎Waltham, MA‎, U.S) according to manufacturer’s instructions.

Transformed *E. Coli* were grown in 5 ml LB medium supplemented with kanamicin, plasmids were purified by Qiaprep Miniprep columns (Qiagen GmbH, Hilden, Germany) and checked for the presence of the expected inserts by 25 cycles-PCR amplification using BV-BC paired primers. A total of 50 plasmids were examined for each sample. Positive samples were prepared using the BigDye™ Terminator Cycle Sequencing Ready Reaction Kit (Thermo Fisher, ‎Waltham, MA‎, U.S) according to the manufacturer's instructions and an M13 forward primer, then sequences were purified with the Magnesil® kit (Promega, Madison, WI, US). The samples were then denatured in formamide and analyzed on an Applied Biosystem 3130 Genetic Analyser using Sequencing-analysis software (Thermo Fisher, ‎Waltham, MA‎, U.S). V-D-J junction analysis was done using IMGT/Junction Analysis tool, available at IMGT web site (ImMunoGeneTics, [www.imgt.com](http://www.imgt.com)) (Table S3). DNA sequence was translated into protein sequence through the ExPASy Proteomics Server ([http://au.expasy.org/).](http://au.expasy.org/).%20) The number and the sequences obtained are displayed in Table S4 and S5 for BV21 and BV28, respectively.

**1.8 DNA extraction and HLA-A genotyping**

Genomic DNA from whole blood was extracted by QIAamp DNA Mini kits (Qiagen GmbH, Hilden, Germany) and 0.1 micrograms of purified genomic DNA were used for HLA-DRB1 exon PCR amplification. After PCR amplification, HLA-A molecular typing - was performed by a reverse hybridization method using the INNO-LiPA HLA-A kit (Fujirebio, Tokyo, Japan), following manufacturer’s instructions. Interpretation of hybridization of HLA-A probes was made by use of LiRAS software (Fujirebio, Tokyo, Japan), to predict one-digit HLA.

***1.9 In silico* modelling**

The homology modelling algorithm MODELLER v9.10^5^ as implemented in Discovery Studio 4.0 (Dassault Systèmes) was used to generate the computational model structure of TRBV21*. The three-dimensional structure of a 3UTT (1E6) bound to an HLA-A*0201-restricted glucose-sensitive preproinsulin peptide (PDB code: 3UTT)^6^, showing 70.0% sequence identity, was used as template (Figure S2). The best-ranked model based on PDF (Probability Density Function) was selected and the quality of the structure was assessed by PROCHECK^7^ and VERIFY3D^8^ (Figure S3 and S4). The structure of the human major histocompatability (MHC) class I molecule HLA-A*0301 (HLA-A3) in complex with a peptide (KLIETYFSK) from proteolipid protein^9^, was used as interaction partner for the modelled TRBV21*. The ternary complex 1BD2^10^ was used as reference for the relative orientation of the interacting structures. Following the replacement of each residue by Glycine (Gly), the peptide backbone was then used as a template to build putative epitope peptides by side chain construction and CHARMM energy minimization^11^. This process was automatically performed by the Grow Scaffold module in Discovery Studio 4.0 by identifying the top ranking residue in each position. After calculating and scoring, the best peptide to act as ligand was selected for further analyses^12-14^.

**1.10 Sequence alignment and similarity analysis**

An alignment search of the specific peptide sequence identified by computational modeling to a specific database of bacterial peptides was performed to find the closest matching. We used the Basic Local Alignment Search Tool (BLAST) program.^15^ from National Center for Biotechnology Information (NCBI). The top scoring alignment (*i.e.* lowest E value) with an alignment length of 6 or more continuous amino-acids in a string of 9 was used as the best match.

**2 Supplementary references**

1. Ria F, Gallard A, Gabaglia CR, Guéry J-C, Sercarz EE, Adorini L. Selection of similar naive T cell repertoires but induction of distinct T cell responses by native and modified antigen. J Immunol. 2004; 172: 3447–53.
2. Ria F, Penitente R, De Santis M, Nicolò C, Di Sante G, Orsini M, et al. Collagen-specific T-cell repertoire in blood and synovial fluid varies with disease activity in early rheumatoid arthritis. Arthritis Res Ther. 2008; 10:R135.
3. Di Sante G, Tolusso B, Fedele AL, Gremese E, Alivernini S, Nicolò C, et al. Collagen Specific T-Cell Repertoire and HLA-DR Alleles: Biomarkers of Active Refractory Rheumatoid Arthritis. EBioMedicine. 2015; 2: 2037–45.
4. Gorochov, G., A. U. Neumann, A. Kereveur, C. Parizot, T. Li, C. Katlama, M. Karmochkine, G. Raguin, B. Autran, and P. Debre. Perturbation of CD4+ and CD8+ T-cell repertoires during progression to AIDS and regulation of the CD4+ repertoire during antiviral therapy. Nat Med 1998; 4: 215-21.
5. Eswar N, Webb B, Marti-Renom MA, Madhusudhan MS, Eramian D, Shen M, et al. Comparative Protein Structure Modeling Using Modeller. Current Protocols in Bioinformatics. 2006; 15: 5.6.1-5.6.30.
6. Bulek AM, Cole DK, Skowera A, Dolton G, Gras S, Madura F, et al. Structural basis for the killing of human beta cells by CD8+ T cells in type 1 diabetes. Nature Immunology. 2012; 13: 283–9.
7. Laskowski RA, MacArthur MW, Moss DS, Thornton JM. PROCHECK: a program to check the stereochemical quality of protein structures. Journal of Applied Crystallography. 1993; 26: 283–91.
8. Eisenberg D, Lüthy R, Bowie JU. [20] VERIFY3D: Assessment of protein models with three-dimensional profiles. In: Methods in Enzymology [Internet]. Elsevier; 1997 [cited 2018 Nov 17]. p. 396–404. Available from: <http://linkinghub.elsevier.com/retrieve/pii/S0076687997770228>
9. McMahon RM, Friis L, Siebold C, Friese MA, Fugger L, Jones EY. Structure of HLA-A*0301 in complex with a peptide of proteolipid protein: insights into the role of HLA-A alleles in susceptibility to multiple sclerosis. Acta Crystallographica Section D Biological Crystallography. 2011; 67: 447–54.
10. Ding Y-H, Smith KJ, Garboczi DN, Utz U, Biddison WE, Wiley DC. COMPLEX BETWEEN HUMAN T-CELL RECEPTOR B7, VIRAL PEPTIDE (TAX) AND MHC CLASS I MOLECULE HLA-A 0201. 1998 Aug 19 [cited 2018 Nov 17]; Available from: <ftp://ftp.wwpdb.org/pub/pdb/data/structures/divided/pdb/bd/pdb1bd2.ent.gz>.
11. Brooks BR, Brooks CL, Mackerell AD, Nilsson L, Petrella RJ, Roux B, et al. CHARMM: The biomolecular simulation program. Journal of Computational Chemistry. 2009 Jul 30;30(10):1545–614.
12. Bissantz, C.; Kuhn, B.; Stahl, M., A Medicinal Chemist's Guide to Molecular Interactions. J. Med. Chem. 2010, 53, 5061-5084.
13. Thompson JD, Higgins DG, Gibson TJ. CLUSTAL W: improving the sensitivity of progressive multiple sequence alignment through sequence weighting, position-specific gap penalties and weight matrix choice. Nucleic Acids Res. 1994; 22: 4673–80.
14. Robert X, Gouet P. Deciphering key features in protein structures with the new ENDscript server. Nucleic Acids Research. 2014; 42(W1):W320–4.
15. Camacho C, Coulouris G, Avagyan V, Ma N, Papadopoulos J, Bealer K, Madden TL BLAST+: architecture and applications.BMC Bioinformatics 2009; 10: 421.

# Supplementary Figures

**
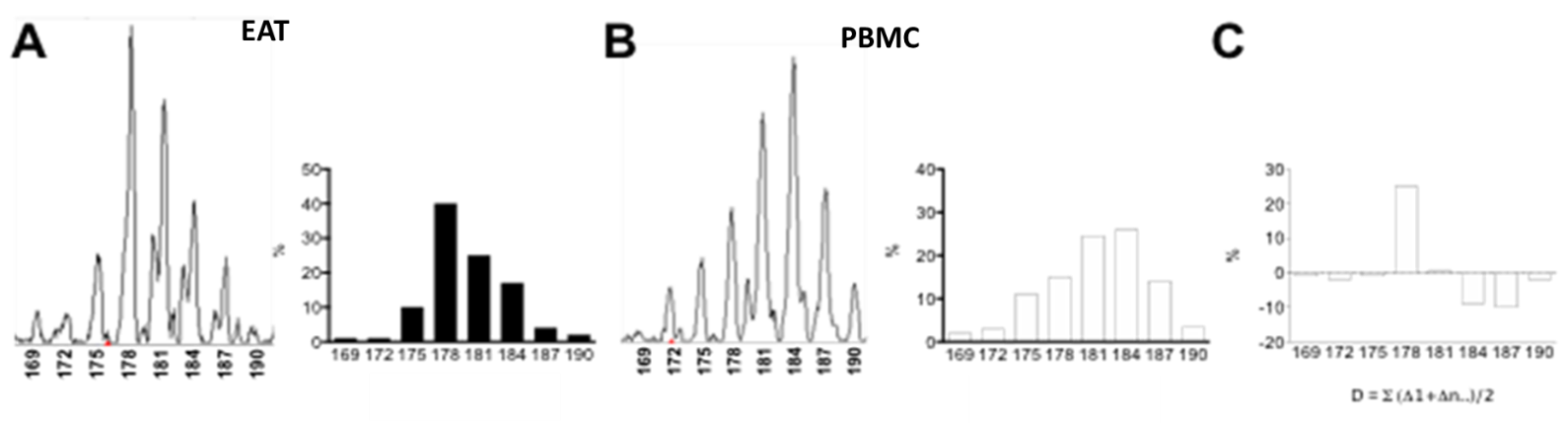
**

**Figure S1. Method for quantitative analysis of TCR repertoire perturbations**. Exemplificative experiment for TRBV21 from a NSTEMI patient. The CDR3 length profile is translated into a p distribution pBV21(i) for each peak (i=169–190), for EAT (A) and PBMC (B) samples, as a function of the area under the curve, with normalization so that Σ of pBV21 (i) = 100%. C) The extent of perturbation ΔBV21(i) for each peak (i) is computed by the difference between pBV21(i) values from the EAT sample and pBV21(i) values from a reference distribution obtained from analysis of corresponding PBMC sample. The perturbation (D) of BV21 is then calculated as Σ ΔBV21(i)/2. Modified from *Gorochov et al. Nat Med 1998*^4^.


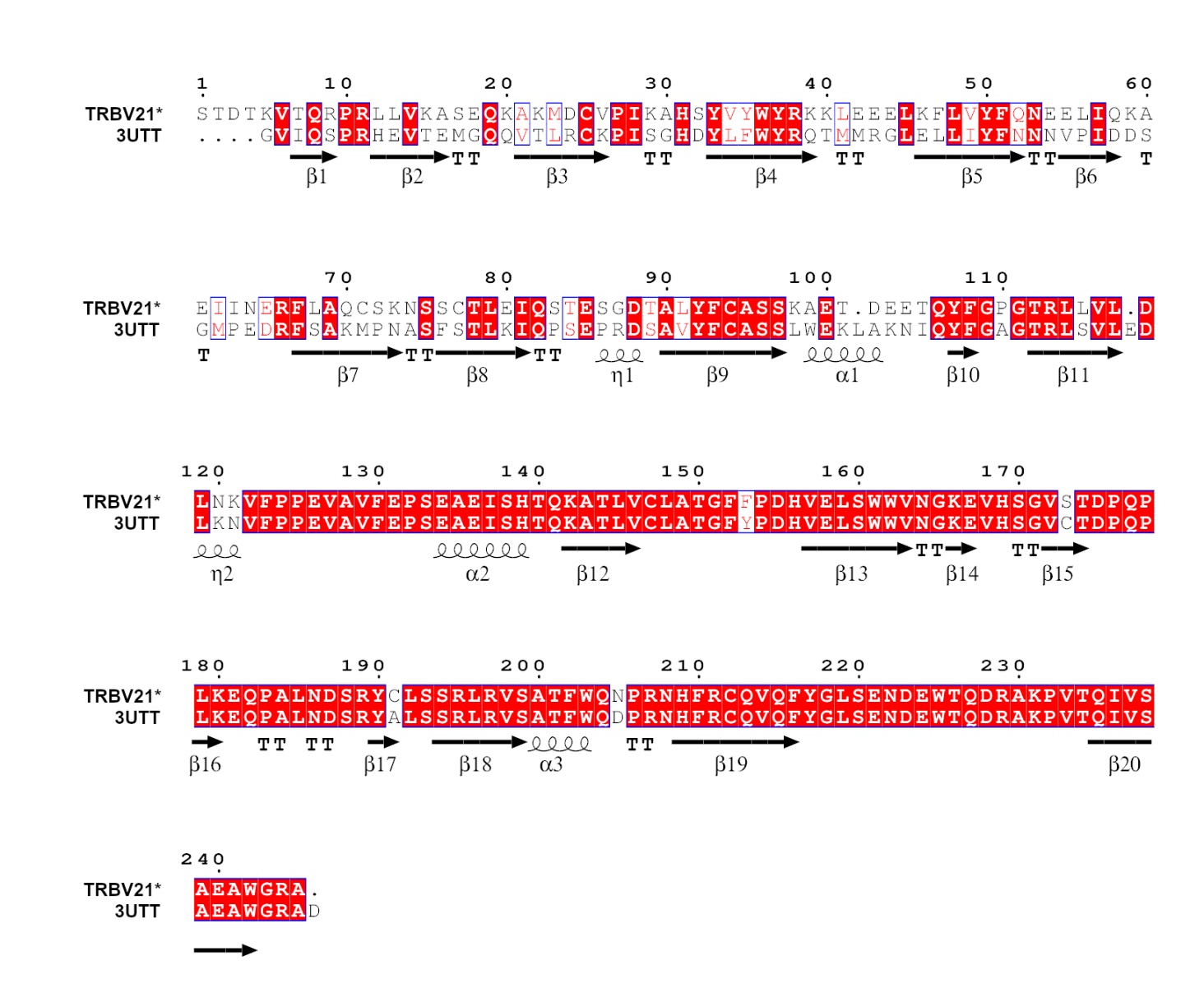


**Figure S2.** Sequence alignment of TRBV21* with 3UTT. The alignment was generated by ClustalW (<https://www.genome.jp/tools-bin/clustalw>)^13^ and secondary structure elements are shown. The Figure was prepared using ESPript (<http://espript.ibcp.fr/ESPript/ESPript/> ^14^.


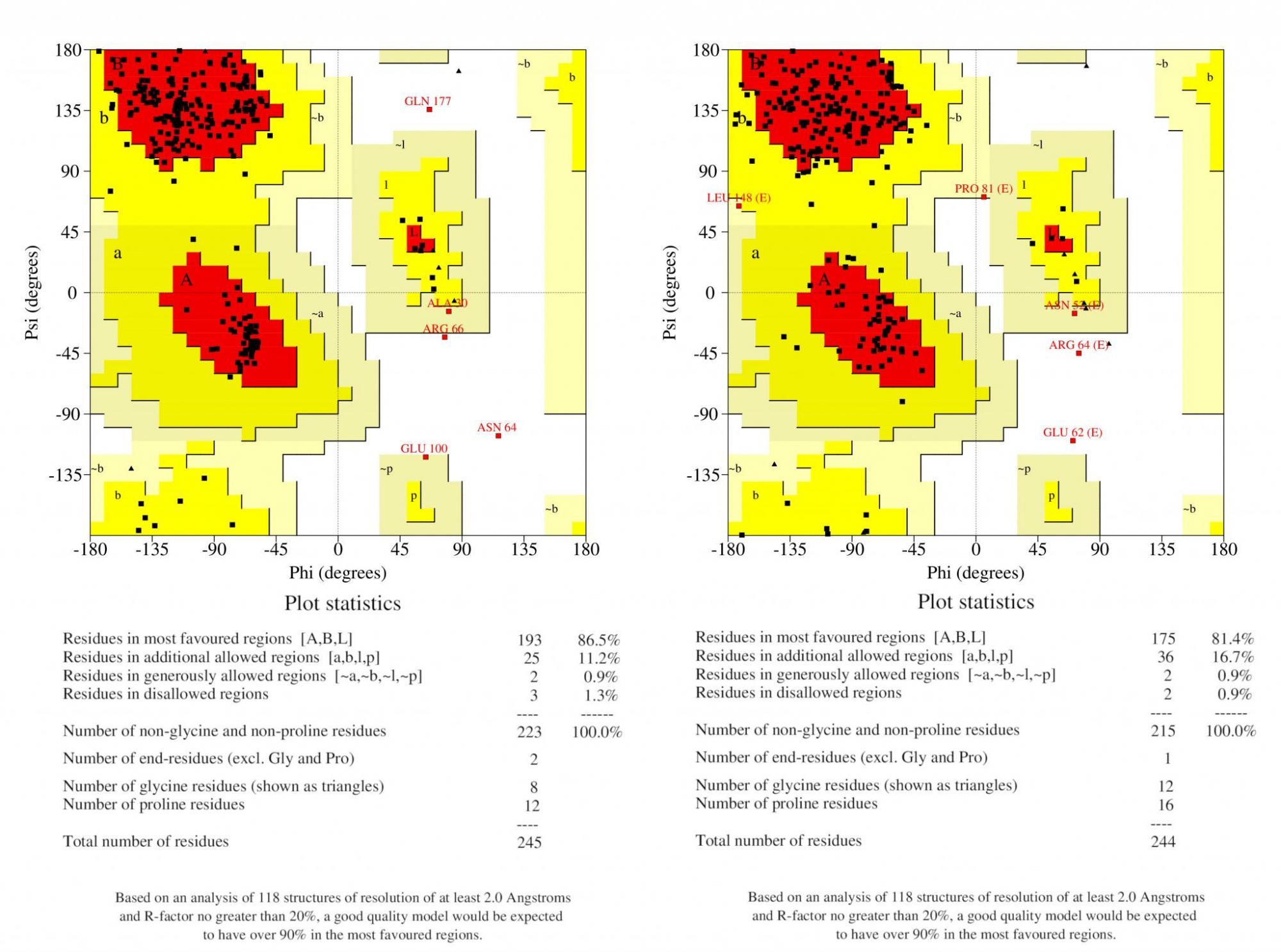


**Figure S3.** Stereochemical assessment by PROCHECK. Ramachandran plot of TRVB model structure (A) and 3UTT crystallographic structure (B).


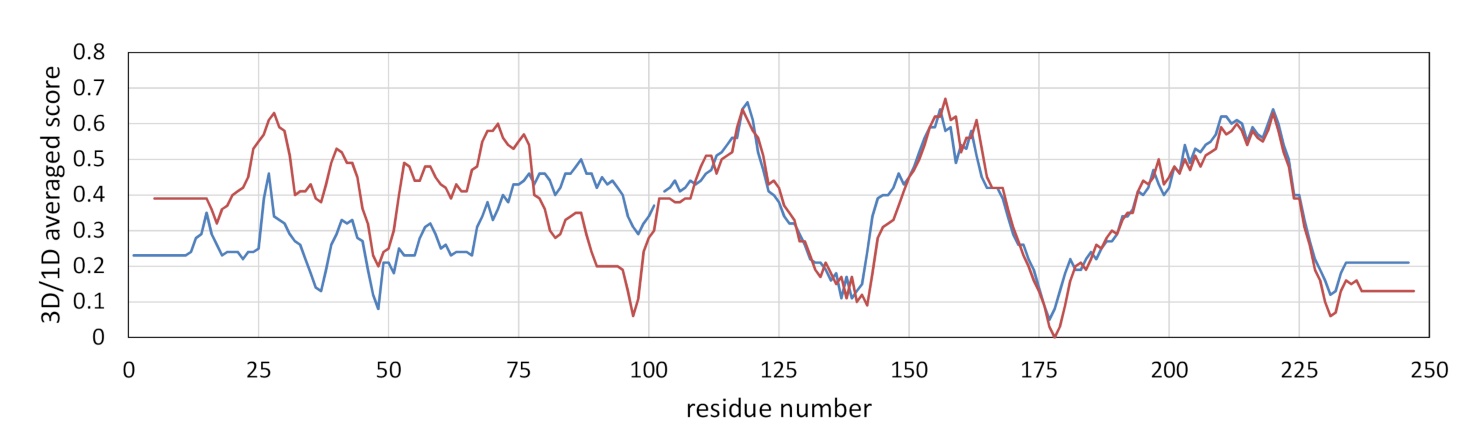


**Figure S4.** Structure quality assessment by VERIFY3D. The plot of TRBV model structure (blue) in comparison with 3UTT crystallographic structure (red) is shown.

**
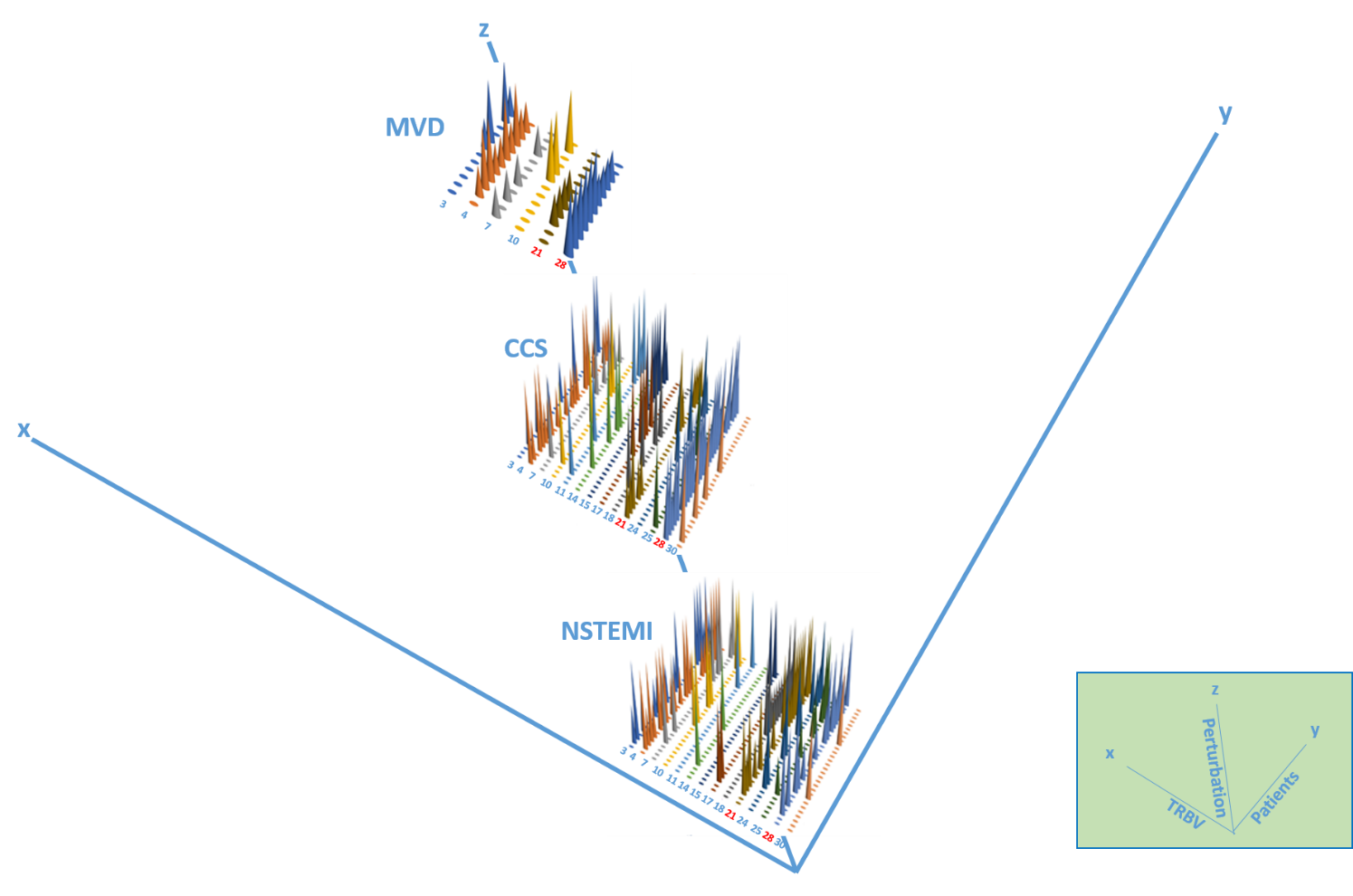
**

**Figure S5.** Quantitative analysis of TCR repertoire perturbations. The TCR repertoire analysis has been performed through spectratyping technique dissecting more than 75% of the whole TCR repertoire. EAT samples are compared with the respective PBMC. Each peak (cones) represents a perturbation (D) of the Gaussian expressed as percentage of one TRBV-TRBC rearrangements for each patient of the three groups enrolled: NSTEMI, Non ST-Elevation Myocardial Infarction, CCS, Chronic Coronary Syndrome, and MVD, Mitral Valve Disease. The graphs show that each patient presents mainly exclusive repertoire, although it is possible to highlight a high frequency of peaks of perturbation for TRBV21 and TRBV28 for NSTEMI and CCS patients, respectively.

**
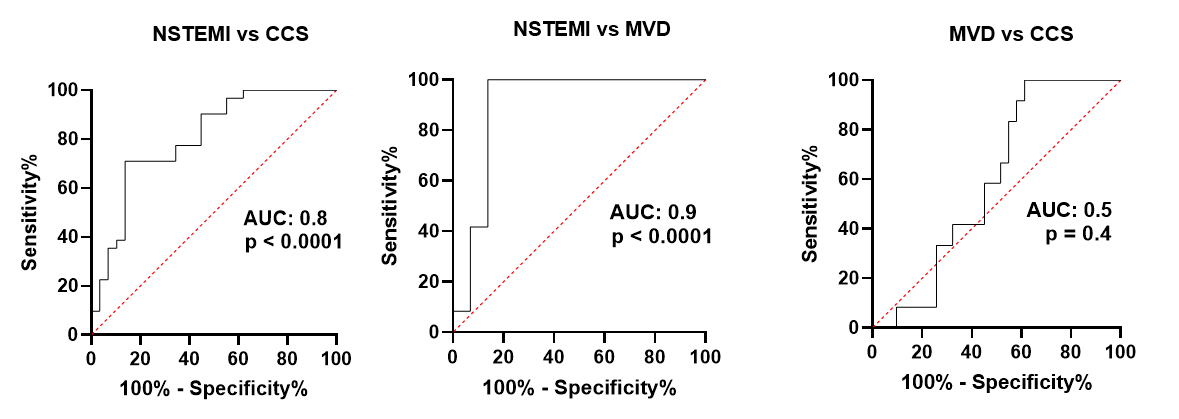
**

**Figure S6.** Receiving Operating Character (ROC) curves for TRBV21 perturbation levels (D%).

**Figure S7.** Quantitative analysis of TRBV21 perturbation in NSTEMI patients (C). Twelve NSTEMI patients with high perturbation of TRBV21 (D>10%) are displayed in the 3D graph where PBMC (blue cones) and EAT (red cylinders) samples are individually showed comparing the expansions (y=%) of each peak (x=CDR3 length), highlighting that the most frequently perturbed peak is the 178 bases length (red number).

**
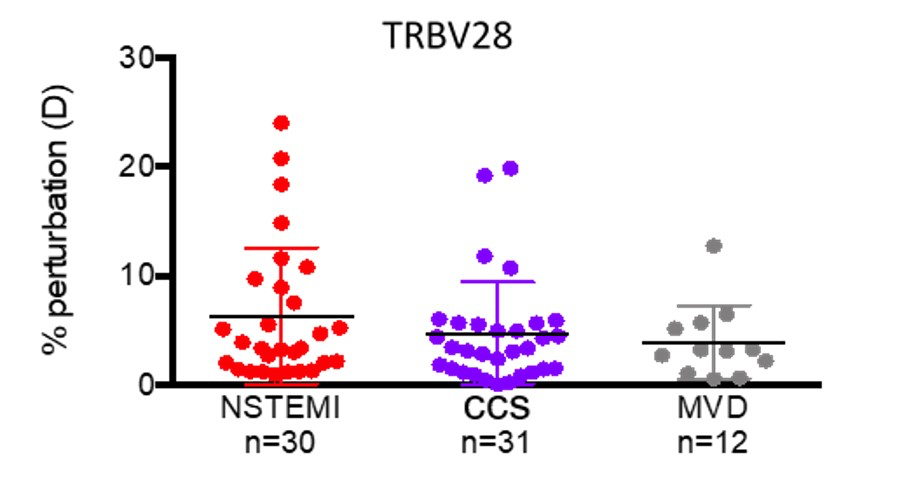
**

**Figure S8.** Quantitative analysis of TCR repertoire perturbations (D) of TRBV28 in EAT samples as compared with PBMC. No differences were observed in average perturbations (D) TRBV28 among groups.

# Supplementary Tables

**Table S1.** Demographic and clinical characteristics of the [study](https://www.sciencedirect.com/topics/medicine-and-dentistry/population-research) population

|  | **MVD**  **(n=12)** | **CCS**  **(n=34)** | **NSTEMI**  **(n=32)** | **p-value** |
| --- | --- | --- | --- | --- |
| **DEMOGRAPHIC CHARACTERISTICS** | | | | |
| Age, years | 70.5 ± 7 | 69.6 ± 8 | 70.2 ± 8 | 0.93 |
| Sex, male/female | 6/6 | 24/10 | 25/7 | 0.19 |
| BMI (kg/m^2^) | 25±5 | 27±4 | 28±4 | 0.14 |
| Time from index acute event  to CABG, days | NA | NA | 10.1 ± 3.2 | NA |
| **CARDIOVASCULAR RISK FACTORS** | | | | |
| Hypertension | 5 (42) | 26 (76) | 22 (69) | 0.09 |
| Dyslipidemia | 0 (0) | 18 (53) | 16 (50) | <0.01 |
| Smoke | 4 (33) | 18 (53) | 19 (59) | 0.30 |
| Family history of IHD | 1 (8) | 8 (24) | 11 (34) | 0.20 |
| Obesity | 2 (17) | 6 (18) | 9 (28) | 0.65 |
| Diabetes | 2 (17) | 9 (26) | 15 (47) | 0.10 |
| **PREVIOUS CORONARY ARTERY DISEASE HISTORY** | | | | |
| Previous STEMI/NSTEMI | 0 (0) | 13 (38) | 13 (41) | 0.02 |
| Previous PCI | 0 (0) | 3 (9) | 3 (9) | 0.73 |
| Previous CABG | 0 (0) | 0 (0) | 0 (0) | NA |
| **MEDICATIONS (AT THE TIME OF BLOOD SAMPLING)** | | | | |
| Aspirin | 0 (0) | 11 (32) | 8 (25) | 0.06 |
| P2Y_12_ receptor inhibitors | 0 (0) | 5 (15) | 6 (19) | 0.31 |
| ACE inhibitors | 7 (58) | 12 (35) | 16 (50) | 0.29 |
| ARBs | 3 (25) | 9 (26) | 8 (25) | 1.00 |
| Calcium-channel blockers | 1 (8) | 12 (35) | 6 (19) | 0.13 |
| Statins | 0 (0) | 23 (68) | 24 (75) | <0.01 |
| β-Blockers | 7 (58) | 25 (74) | 21 (66) | 0.57 |
| Diuretic agents | 8 (67) | 15 (44) | 14 (44) | 0.35 |
| Oral antidiabetic drugs | 1 (8) | 5 (15 | 8 (25) | 0.44 |
| Insulin | 1 (8) | 3 (9) | 8 (25) | 0.18 |
| LMWH | 0 (0) | 2 (6) | 3 (9) | 0.70 |
| **LABORATORY ASSAY** | | | | |
| cTnI >0.004 ng/ml | NA | NA | 33 (100) | NA |
| Hemoglobin, g/dl | 13 ± 2 | 13 ± 2 | 13 ± 1 | 0.85 |
| Lymphocyte count, 10^9^/l | 3 ± 1 | 2 ± 2 | 3 ± 1 | 0.43 |
| Platelets, 10^3^/ml | 186 ± 60 | 222 ± 60 | 219 ± 55 | 0.19 |
| Glycemia, mg/dl | 106 ± 21 | 114 ± 40 | 115 ± 31 | 0.82 |
| Total cholesterol, mg/dl | 144.9 ± 30 | 185.2 ± 39 | 179 ± 4 | 0.02 |
| LDL, mg/dl | 101.7 ± 26 | 115 ± 32 | 112 ± 40 | 0.53 |
| HDL, mg/dl | 49 ± 12 | 50.8 ± 16 | 42.8 ± 11 | 0.09 |
| Triglycerides, mg/dl | 142 ± 40 | 168 ± 70 | 169 ± 82 | 0.52 |
| Creatinine, mg/dl | 0.7 ± 0.1 | 0.8 ± 0.3 | 1.11 ± 0.4 | 0.09 |
| ESR, mm/h | 9 ± 8 | 14 ± 12 | 20 ± 11 | 0.22 |
| hs-CRP, mg/l | 3.2 ± 3 | 6.4 ± 8 | 24.6 ± 9 | 0.002 |
| **IN-HOSPITAL MANAGEMENT** | | | | |
| Multivessel disease | NA | 28 (82) | 31 (97) | <0.001* |
| Culprit coronary artery   - LCA - RCA | NA  NA | NA  NA | 20 (63)  12 (37) | NA  NA |
| LVEF≥50% | 12 (100) | 24 (71) | 22 (69) | 0.06 |

Values are mean ± SD, numbers (%), or median (interquartile range). *p value for CCS vs NSTEMI = 0.106

ACE=[angiotensin-converting enzyme](https://www.sciencedirect.com/topics/medicine-and-dentistry/dipeptidyl-carboxypeptidase); NSTEMI=Non-ST-Elevation Myocardial Infarction; ARBs=[angiotensin II receptor blockers](https://www.sciencedirect.com/topics/medicine-and-dentistry/angiotensin-receptor-antagonist); CABG=[coronary artery bypass grafting](https://www.sciencedirect.com/topics/medicine-and-dentistry/coronary-artery-bypass-graft); cTnI=[cardiac troponin](https://www.sciencedirect.com/topics/medicine-and-dentistry/cardiac-troponin) I; CTRL=control; ESR=[erythrocyte sedimentation rate](https://www.sciencedirect.com/topics/medicine-and-dentistry/erythrocyte-sedimentation-rate); HDL=[high-density lipoprotein](https://www.sciencedirect.com/topics/nursing-and-health-professions/high-density-lipoprotein); hs-CRP=high-sensitivity [C-reactive protein](https://www.sciencedirect.com/topics/medicine-and-dentistry/c-reactive-protein); IHD=[ischemic heart disease](https://www.sciencedirect.com/topics/medicine-and-dentistry/ischemic-heart-disease); LCA=left coronary artery; LDL=[low-density lipoprotein](https://www.sciencedirect.com/topics/nursing-and-health-professions/low-density-lipoprotein); LVEF=[left ventricular ejection fraction](https://www.sciencedirect.com/topics/medicine-and-dentistry/heart-left-ventricle-ejection-fraction); NA=not applicable; RCA=right coronary artery; CCS = [chronic](https://www.sciencedirect.com/topics/nursing-and-health-professions/stable-angina-pectoris) coronary syndrome.

|  | **CCS**  **(Mean ± SD)** | **NSTEMI**  **(Mean ± SD)** | **p-value** |  |
| --- | --- | --- | --- | --- |
| Adiponectin (Acrp30) | 1,180 ± 0,057 | 1,232 ± 0,029 | 0,0120 | |
| Angiogenin (ANG) | 0,413 ± 0,026 | 0,587 ± 0,023 | <0,0001 | |
| Apolipoprotein A1 (Apo A1) | 0,262 ± 0,021 | 0,315 ± 0,028 | 0,0079 | |
| Complement component 5/5a (C5/C5a) | 0,019 ± 0,003 | 0,040 ± 0,0004 | ns | |
| C-C Motif Chemokine Ligand 5 (CCL5) | 0,017 ± 0,0003 | 0,035 ± 0,002 | ns | |
| Cluster of Differentiation 14 (CD14) | 0,041 ± 0,003 | 0,082 ± 0,005 | ns | |
| Cluster of Differentiation 31 (CD31) | 0,192 ± 0,011 | 0,423 ± 0,017 | <0,0001 | |
| Cluster of Differentiation 40 Ligand (CD40L) | 0,067 ± 0,005 | 0,093 ± 0,003 | ns | |
| Complement Factor D (CFD) | 0,057 ± 0,004 | 0,121 ± 0,010 | 0,0005 | |
| Chitinase-3-like protein 1 (CHI3L1) | 0,080 ± 0,005 | 0,338 ± 0,029 | <0,0001 | |
| C-Reactive Protein (CRP) | 0,058 ± 0,003 | 0,521 ± 0,035 | <0,0001 | |
| C-X-C Motif Chemokine Ligand 10 (CXCL10) | 0,013 ± 0,0002 | 0,042 ± 0,004 | ns | |
| Dipeptidyl Peptidase 4 (DPPIV) | 0,038 ± 0,004 | 0,058 ± 0,005 | ns | |
| Extracellular matrix metalloproteinase inducer (EMMPRIN) | 0,196 ± 0,006 | 0,258 ± 0,005 | 0,0009 | |
| Endoglin (ENG) | 0,055 ± 0,001 | 0,136 ± 0,022 | <0,0001 | |
| Fibroblast Growth Factor 19 (FGF-19) | 0,027 ± 0,002 | 0,052 ± 0,0002 | ns | |
| Intercellular Adhesion Molecule 1 (ICAM-1) | 0,030 ± 0,002 | 0,065 ± 0,007 | ns | |
| Interleukin-8 (IL-8) | 0,023 ± 0,001 | 0,044 ± 0,001 | ns | |
| Interleukin-17 (IL-17) | 0,043 ± 0,004 | 0,143 ± 0,002 | <0,0001 | |
| Interleukin-33 (IL-33) | 0,023 ± 0,004 | 0,121 ± 0,008 | <0,0001 | |
| Macrophage Migration Inhibitory Factor (MIF) | 0,119 ± 0,0004 | 0,148 ± 0,008 | ns | |
| Macrophage Inflammatory Protein-3 beta (MIP3β) | 0,016 ± 0,002 | 0,040 ± 0,003 | ns | |
| Matrix metalloprotease-9 (MMP-9) | 0,216 ± 0,008 | 0,322 ± 0,008 | <0,0001 | |
| Myeloperoxidase (MPO) | 0,039 ± 0,004 | 0,114 ± 0,002 | <0,0001 | |
| Neutrophil gelatinase-associated lipocalin (NGAL) | 0,117 ± 0,003 | 0,336 ± 0,042 | <0,0001 | |
| Osteopontin (OPN) | 0,030 ± 0,0003 | 0,037 ± 0,005 | ns | |
| Resistin (RETN) | 0,028 ± 0,0001 | 0,220 ± 0,007 | <0,0001 | |
| Retinol-binding protein-4 (RBP-4) | 0,056 ± 0,004 | 0,150 ± 0,007 | <0,0001 | |
| Stromal cell-derived factor 1 (SDF1α) | 0,030 0,0002 | 0,041 ± 0,001 | ns | |
| Sex Hormone Binding Globulin (SHBG) | 0,025 ± 0,003 | 0,044 ± 0,006 | ns | |
| T cell immunoglobulin and mucin domain-containing protein 3 (TIM-3) | 0,020 ± 0,0005 | 0,054 ± 0,009 | ns | |
| Thrombospondin 1 (TSP-1) | 0,025 ± 0,004 | 0,061 ± 0,003 | ns | |
| Urokinase-type plasminogen activator receptor (uPAR) | 0,017 ± 0,0005 | 0,051 ± 0,004 | ns | |
| Vascular cell adhesion protein 1 (VCAM1) | 0,020 ± 0,001 | 0,059 ± 0,005 | ns | |
| Vitamin D-binding protein (VDB) | 0,122 ± 0,013 | 0,427 ± 0,025 | <0,0001 | |

**Table S3**. Sequences of specific primers used for RT-PCR.

| Alias name | SEQUENCE | TR gene |
| --- | --- | --- |
| hcβ1α | GGGTGTGGGAGATCCTGC | TRBC1a |
| hvβ1 | CCGCACAACAGTTCCCTGACTTGC | TRBV9 |
| hvβ 3 | CGCTTCTCCCTGATTCTGGAGTCC | TRBV28 |
| hvβ 4 | TTCCCATCAGCCGCCCAAACCTAA | TRBV29 |
| hvβ 5 | GATCAAAACGAGAGGACAGC | TRBV5 |
| hvβ 6a | GATCCAATTTCAGGTCATACTG | TRBV7 |
| hvβ 6b1 | CAGGGCCAGAGTTTCTGAC | TRBV7-2 |
| hvβ 6b2 | CAGGGCTCAGAGGTTCTGAC | TRBV7-4 |
| hvβ 7 | CCTGAATGCCCCAACAGCTCT | TRBV4 |
| hvβ 8 | GGTACAGACAGACCATGATGC | TRBV12 |
| hvβ 9 | TTCCCTGGAGCTTGGTGACTCTGC | TRBV3 |
| hvβ 10 | CCACGGAGTAGGGGACACAGCAC | TRBV21 |
| hvβ 11 | GTCAACAGTCTCCAGAATAAGG | TRBV25 |
| hvβ 12 | TCCYCCTCACTCTGGAGTC | TRBV10 |
| hvβ 13a | GTATCGACAAGACCCAGGCA | TRBV6 |
| hvβ 13b | AGGCTCATCCATTATTCAAATAC | TRBV6-4 |
| hvβ 14 | GGGCTGGGCTTAAGGCAGATCTAC | TRBV27 |
| hvβ 15 | CAGGCACAGGCTAAATTCTCCCTG | TRBV24 |
| hvβ 16 | GCCTGCAGAACTGGAGGATTCTGG | TRBV14 |
| hvβ 17 | TCCTCTCACTGTGACATCGGCCCA | TRBV19 |
| hvβ 18 | CTGCTGAATTTCCCAAAGAGGGCC | TRBV18 |
| hvβ 20 | TGCCCCAGAATCTCTCAGCCTCCA | TRBV30 |
| hvβ 21 | GGAGTAGACTCCACTCTCAAG | TRBV11 |
| hvβ 22 | GATCCGGTCCACAAAGCTGG | TRBV2-1 |
| hvβ 23 | ATTCTGAACTGAACATGAGCTCCT | TRBV13 |
| hvβ 24 | GACATCCGCTCACCAGGCCTG | TRBV15 |
| hJβ 1.1 | TCTGGTGCCTTGTCCAAAGAAAGC | TRBJ1.1 |
| hJβ 1.2 | CCTGTCCCCGAACCGAAGGTGTA | TRBJ1.2 |
| hJβ 1.3 | CCAACTTCCCTCTCCAAAATATAT | TRBJ1.3 |
| hJβ 1.4 | CTGGGTTCCACTGCCAAAAAACAG | TRBJ1.4 |
| hJβ 1.5 | TCGAGTCCCATCACCAAAATGCTG | TRBJ1.5 |
| hJβ 1.6 | CCTGGTCCCATTCCCAAAGTGGAG | TRBJ1.6 |
| hJβ 2.1 | CCGTGTCCCTGGCCCGAAGAACTG | TRBJ2.1 |
| hJβ 2.2 | CTAGAGCCTTCTCCAAAAAACAGC | TRBJ2.2 |
| hJβ 2.3 | CTAGAGCCTTCTCCAAAAAACAGC | TRBJ2.3 |
| hJβ 2.4 | GGGTCCCGGCGCCGAAGTACTGAA | TRBJ2.4 |
| hJβ 2.5 | CGCGTGCCTGGCCCGAAGTACTGG | TRBJ2.5 |
| hJβ 2.6 | GCTGCCGGCCCCGAAAGTCAGGAC | TRBJ2.6 |
| hJβ 2.7 | TGGTGCCCGGCCCGAAGTACTGCT | TRBJ2.7 |

TRBV-, TRBC- and TRBJ- primer sequences were deduced from ImMunoGeneTics (IMGT) databases. Here we display both the gene and the alias name reported in the nomenclature of IMGT/ Collier-de-Perles. TRBJ primers when needed were purchased with the labeling with a fluorescent Dye (6-carboxyfluorescein or FAM).

**Table S4.** CDR3 region sequencing for TRBV21 and TRBV28.

| **Group** | **Sample**  **code** | **Primers** | | **Plasmids** | **Insert** | **In frame** |
| --- | --- | --- | --- | --- | --- | --- |
|  |  | **Forward** | **reverse** |  |  |  |
| NSTEMI | 16 EAT | TRBV21 | TRBC | 50 | 5 | 23 |
| NSTEMI | 16 PBMC | TRBV21 | TRBC | 50 | 41 | 1 |
| NSTEMI | 17 EAT | TRBV21 | TRBC | 50 | 40 | 8 |
| NSTEMI | 17 PBMC | TRBV21 | TRBC | 20 | 14 | 1 |
| NSTEMI | 21 EAT | TRBV21 | TRBC | 50 | 15 | 3 |
| NSTEMI | 24 EAT | TRBV21 | TRBC | 50 | 40 | 25 |
| NSTEMI | 24 EAT | TRBV21 | TRBJ2.5 | 20 | 4 | 4 |
| NSTEMI | 24 PBMC | TRBV21 | TRBC | 20 | 13 | 4 |
| NSTEMI | 38 EAT | TRBV21 | TRBC | 50 | 30 | 1 |
| NSTEMI | 38 PBMC | TRBV21 | TRBC | 50 | 27 | 2 |
| NSTEMI | 46 EAT | TRBV21 | TRBC | 50 | 21 | 2 |
| NSTEMI | 66 EAT | TRBV21 | TRBC | 50 | 18 | 5 |
| NSTEMI | 85 EAT | TRBV21 | TRBC | 50 | 20 | 4 |
| CCS | 87 EAT | TRBV21 | TRBC | 50 | 15 | 4 |
| CCS | 8 EAT | TRBV21 | TRBJ2.5 | 50 | 15 | 1 |
| CCS | 9 EAT | TRBV21 | TRBJ2.5 | 50 | 22 | 3 |
| CCS | 9 EAT | TRBV28 | TRBC | 50 | 20 | 16 |
| CCS | 10 EAT | TRBV28 | TRBC | 50 | 20 | 18 |
| CCS | 22 EAT | TRBV28 | TRBC | 50 | 12 | 4 |
| NSTEMI | 14 EAT | TRBV28 | TRBC | 50 | 20 | 2 |
| NSTEMI | 21 EAT | TRBV28 | TRBC | 50 | 36 | 10 |
| NSTEMI | 21 EAT | TRBV28 | TRBJ2.1 | 20 | 15 | 8 |
| NSTEMI | 38 EAT | TRBV28 | TRBJ2.1 | 20 | 10 | 9 |
| Total | | | | 1000 | 473 | 158 |

**Table S5.** Sequences of CDR3 region from samples with perturbated TRBV21. CASSKA ETDE ETQYFGPGTRL

| Group | Sample  code | VDJC length | **TRBV21-D-J** segment (CDR3 region) | TRBJ |
| --- | --- | --- | --- | --- |
| NSTEMI | 16 EAT | 169 | C A R **R G** Q P N Y G Y T F G S G T | 1.1 |
|  |  | 169 | C A S S K **R** G P N E Q F F G P G T R L | 2.1 |
|  |  | 170 | C A S **R G G T** N T E A F F G Q G T | 1.1 |
|  |  | 170 | C A S **R G G T** N T E A F F G Q G T R | 1.1 |
|  |  | 172 | C A S **R G G T** N T E A F F G Q G T R | 1.1 |
|  |  | 172 | C A S **R G G T** N T E A F F G Q G T R | 1.1 |
|  |  | 172 | C A S S **K R G P** N E Q F F G P G T R | 2.1 |
|  |  | 172 | C A S R G **Q G V T** N E Q F F G P G T R L | 2.1 |
|  |  | 172 | C A R S K **F R G** N Q P Q H F G D G T R | 1.5 |
|  |  | 175 | C A S S **N A P M** N T E A F F G Q G T R | 1.1 |
|  |  | 175 | C A S S **N A P M** N T E A F F G Q G T R | 1.1 |
|  |  | 175 | C A S **R G Q G V T** N E Q F F G P G T R | 2.1 |
|  |  | 175 | C A S S **N A P M** N T E A F F G Q G T R | 1.1 |
|  |  | 178 | C A S S K A **E T D E** E T Q Y F G P G T R L | 2.5 |
|  |  | 178 | C A S S K A **E T D E** E T Q Y F G P G T R L | 2.5 |
|  |  | 178 | C A S S K **P T R G** G N E Q F F G P G T R | 2.1 |
|  |  | 181 | C A S S K **Y T G S L Q** E T Q Y F G P G T R | 2.5 |
|  |  | 181 | C A S S K **Y T G S L Q** E T Q Y F G P G T R | 2.5 |
|  |  | 181 | C A S S K **Q E L A G S D** P Y E Q Y F G P G | 2.5 |
|  |  | 184 | C A S S S Y **S G R A E S Y** N E Q F F G P G T R L | 2.1 |
|  |  | 184 | C A S S N **E R G N R D T T** Y E Q Y F G P G T R | 2.5 |
|  |  | 184 | C A S S S Y **S G R A E S Y** N E Q F F G P G T R L | 2.1 |
|  |  | 184 | C A S S S Y **S G R A E S Y** N E Q F F G P G T R L | 2.5 |
| NSTEMI | 17 EAT | 172 | C A S **R G G T** N T E A F F G Q G T R L | 1.1 |
|  |  | 175 | C A S S K A **E T D E** E T Q Y F G P G T R L | 2.5 |
|  |  | 178 | C A S S K A **E T D E** E T Q Y F G P G T R L | 2.5 |
|  |  | 178 | C A S S K A **E T D E** E T Q Y F G P G T R L | 2.5 |
|  |  | 178 | C A S S K A **E T D E** E T Q Y F G P G T R L | 2.5 |
|  |  | 178 | C A S S K A **E T D E** E T Q Y F G P G T R L | 2.5 |
|  |  | 178 | C A S S K A **E T D E** E T Q Y F G P G T R L | 2.5 |
|  |  | 178 | C A S S K A **E T D E** E T Q Y F G P G T R L | 2.5 |
| NSTEMI | 21 EAT | 172 | C A S **R G G T** N T E A F F G Q G T R L | 1.1 |
|  |  | 172 | C A S **R G G T** N T E A F F G Q G T R L | 1.1 |
|  |  | 175 | C A S S K **A T G N** Q P Q H F G | 1.5 |
|  |  | 178 | C A S S K A **E T D E** E T Q Y F G P G T R L | 2.5 |
|  |  | 178 | C A S S K A **E T D E** E T Q Y F G P G T R L | 2.5 |
| NSTEMI | 24 EAT | 172 | C A S **R G G T** N T E A F F G Q G T R | 1.1 |
|  |  | 172 | C A S **R G G T** N T E A F F G Q G T R | 1.1 |
|  |  | 172 | C A S S **R A G D** Y E Q Y F G P G T R L | 2.5 |
|  |  | 175 | C A S S P **R D D L Q** Y N E Q F F G P G T | 2.1 |
|  |  | 175 | C A R S K **F R G** N Q P Q H F G D G T R | 1.5 |
|  |  | 175 | C A S S **N A P M** N T E A F F G Q G T R | 1.1 |
|  |  | 175 | C A S S **N A P M** N T E A F F G Q G T R | 1.1 |
|  |  | 175 | C A S S **N A P M** N T E A F F G Q G T R | 1.1 |
|  |  | 175 | C A S S **N A P M** N T E A F F G Q G T R | 1.1 |
|  |  | 175 | C A S S K **PT S** A Y E Q Y F G P G T | 2.5 |
|  |  | 175 | C A S S K **PT S** A Y E Q Y F G P G T | 2.5 |
|  |  | 178 | C A S S K **P H G G D** N E Q F F G P G T R L | 2.1 |
|  |  | 178 | C A S S K A **E T D E** E T Q Y F G P G T R L | 2.5 |
|  |  | 178 | C A S S K A **E T D E** E T Q Y F G P G T R L | 2.5 |
|  |  | 178 | C A S S K A **E T D E** E T Q Y F G P G T R L | 2.5 |
|  |  | 178 | C A S S K A **E T D E** E T Q Y F G P G T R L | 2.5 |
|  |  | 91* | C A S S K A **E T D E** E T Q Y F G P G T R L | 2.5 |
|  |  | 91* | C A S S K A **E T D E** E T Q Y F G P G T R L | 2.5 |
|  |  | 91* | C A S S K A **E T D E** E T Q Y F G P G T R L | 2.5 |
|  |  | 91* | C A S S K A **E T D E** E T Q Y F G P G T R L | 2.5 |
|  |  | 184 | C A S S K V **V R G L A K N I** Q Y F G | 2.5 |
|  |  | 184 | C A S S K V **V R G L A K** **N I** Q Y F G | 2.5 |
|  |  | 184 | C A S S K Q **S G T G F T** Y E Q Y F G P G T | 2.5 |
|  |  | 187 | C A S T **N G E R G G T** Y E Q Y F G P G T | 2.5 |
|  |  | 187 | C A S T **N G E R G G T** Y E Q Y F G P G T | 2.5 |
| NSTEMI | 38 EAT | 181 | C A S S S **S G L A E I** Y E Q Y F G P G T R | 2.1 |
| NSTEMI | 46 EAT | 181 | C A S S Q **G G G F** N E K L F F G S G T Q L | 1.4 |
|  |  | 181 | C A S S Q **G G G F** N E K L F F G S G T Q L | 1.4 |
| NSTEMI | 66 EAT | 172 | C A T S G **Q E G T** Q Y F G P G T R L | 2.5 |
|  |  | 172 | C A T S G **Q E G T** Q Y F G P G T R L | 2.5 |
|  |  | 175 | C A S S Q **G R G M G** E Q Y F G P G T R L | 2.5 |
|  |  | 175 | C A S S **Q D I R** Q P Q H F G D G T R L |  |
|  |  | 175 | C A S S **Q D I R** Q P Q H F G D G T R L | 1.5 |
| NSTEMI | 85 EAT | 175 | C A S S Q **D R A S** E A F F G Q G T R L | 1.1 |
|  |  | 175 | C A S S Q **D R A S** E A F F G Q G T R L | 1.1 |
|  |  | 175 | C A S S Q **V Q G G** Y E Q Y F G P G T R L | 2.5 |
|  |  | 178 | C A S S Q **G R G M G** E Q Y F G P G T R L | 2.5 |
|  |  | 169 | C A S S **Q R D T** Q Y F G P G T R L | 2.5 |
| CCS | 87 EAT | 169 | C A S S **Q R D T** Q Y F G P G T R L | 2.5 |
|  |  | 187 | C A S S Q **D G G T G R V** N Q P Q H F G D G T R L | 1.5 |
|  |  | 187 | C A S S Q **A K T G W L G** E T Q Y F G P G T R L | 2.5 |
|  |  | 187 | C A S S **P R G S S G G S Y** N E Q F F G P G T R L | 2.1 |

*These sequences resulted from the direct cloning of TRBV21-TRBJ2.5

EAT=epicardial adipose tissue

**Table S6.** Sequences of CDR3 region from samples with perturbated TRBV28.

| Sample | Seq. code | VDJC length | **TRBV28-D-J** segment (CDR3 region) | TRBJ |
| --- | --- | --- | --- | --- |
| 9 EAT | 6 | 190 | C A S S Y **Q V** Y E Q Y F G P G T R L | 2.5 |
|  | 3 | 190 | C A S S **Q F** G Y E Q Y F G P G T R L | 2.5 |
|  | 12 | 190 | C A S S **Q F** G Y E Q Y F G P G T R L | 2.5 |
|  | 13 | 190 | C A S S L **L S** G E Q F F G P G T R L | 2.1 |
|  | 17 | 190 | C A S S L **L S** G E Q F F G P G T R L | 2.1 |
|  | 16 | 196 | C A S S L **S R G L** L E Q Y F G P G T R L | 2.5 |
|  | 4 | 199 | C A S S L L G G L D N E Q F F G P G T R L | 2.1 |
|  | 5 | 199 | C A S S L **G L L** N T G E L F F G E G S R L | 2.2 |
|  | 10 | 199 | C A S S R **G P Y R D Y** G Y T F G S G T R L | 1.2 |
|  | 1 | 202 | C A S S **F S T L G** D G A E Q D F G P G T R L | ? |
|  | 8 | 202 | C A T I **D R G T** Y S N Q P Q H F G D G T R L | 1.5 |
|  | 14 | 202 | C A S S L **A R T S V** H N E Q F F G P G T R L | 2.1 |
|  | 2 | 205 | C A S S L **S T S G S S Y** N E Q F F G P G T R L | 2.1 |
|  | 9 | 205 | C A S S L **S T S G S S Y** N E Q F F G P G T R L | 2.1 |
|  | 11 | 205 | C A S S L **S T S G S S Y** N E Q F F G P G T R L | 2.1 |
|  | 15 | 205 | C A S S L **S T S G S S Y** N E Q F F G P G T R L | 2.1 |
| 10 EAT | 18 | 205 | C A S S L **S T S G S S Y** N E Q F F G P G T R L | 2.1 |
|  | 9 | 187 | C A S T E **I D** E Q F F G P G T R L | 2.1 |
|  | 13 | 190 | C A S S **Q F** G Y E Q Y F G P G T R L | 2.5 |
|  | 15 | 193 | C A S K **G G G** Y N E Q F F G P G T R L | 2.1 |
|  | 4 | 193 | C A S S S **R G V** G E L F F G E G S R L | 2.2 |
|  | 19 | 193 | C A S S **F R G** T G E L F F G E G S R L | 2.2 |
|  | 7 | 193 | C A S S **W D R N** Y E Q Y F G P G T R L | 2.7 |
|  | 2 | 196 | C A S S L **K L A G G** E Q Y F G P G T R L | 2.7 |
|  | 3 | 196 | C A S S **T G T G V H** E Q Y F G P G T R L | 2.7 |
|  | 11 | 196 | C A S S **T G T G V H** E Q Y F G P G T R L | 2.7 |
|  | 10 | 196 | C A S S S **T G E G** T E A F F G Q G T R L | 1.1 |
|  | 18 | 196 | C A S T **A G Q F** N T E A F F G Q G T R L | 1.1 |
|  | 5 | 196 | A S S L **R D S Y** Q P Q H F G D G T R L | 2.5 |
|  | 17 | 199 | C A S S L **L G G L D** N E Q F F G P G T R L | 2.1 |
|  | 1 | 199 | C A S S T **Q G E R L D** E Q F F G P G T R L | 2.1 |
|  | 20 | 199 | C A S R V **S G R G A** G E L F F G E G S R L | 2.2 |
|  | 16 | 202 | C A S S G **R G** **G S S Y** N E Q F F G P G T R L | 2.1 |
|  | 12 | 202 | C A S S S **G L R Q G L** E T Q Y F G P G T R L | 2.5 |
| 22 EAT | 8 | 110* | C A S S Q **D R G R G S** Q Y F G P G T | 2.5 |
|  | 5 | 113* | C A S S **G A S G S Q E T** Q Y F G P G T | 2.5 |
|  | 6 | 113* | C A S S **G A S G S Q E T** Q Y F G P G T | 2.5 |
|  | 7 | 113* | C A S S **S Q R R G K E T** Q Y F G P G T | 2.5 |
| 14 EAT | 7 | 192 | C A S S **L R V P** Y E Q Y F G P G T | 2.5 |
|  | 8 | 205 | C A S S **L R V R L A G** L G E L F F G | 2.2 |
| 21 EAT | 1 | 107* | C A S S **R L R** A Y D Q Y F G P G T | 2.5 |
|  | 2 | 107* | C A S S **V F** Y N E Q F F G P G T | 2.1 |
|  | 3 | 106* | C A S S **Q V F** Y N D Q Y F G P G T | 2.1 |
|  | 4 | 193 | C A S S **Q V F** Y N E Q F F G P G T R | 2.1 |
|  | 5 | 193 | C A S S **Q V F** Y N E Q F F G P G T R | 2.1 |
|  | 6 | 193 | C A S S **Q V F** Y N E Q F F G P G T R | 2.1 |
|  | 7 | 190 | C A S **A R Q P L** D Q Y F G P G T R L | 2.5 |
|  | 8 | 215 | C A S S **Q V F** Y N E Q F F G P G T R | 2.1 |
|  | 9 | 193 | C A S S **Q V F** Y N E Q F F G P G T R | 2.1 |
|  | 10 | 107* | C A S S **Q V F** Y N E Q F F G P G T R | 2.1 |
|  | 12 | 107* | C A S S **V F** Y N E Q F F G P G T | 2.1 |
|  | 13 | 107* | C A S S **R L R** A Y D Q Y F G P G T | 2.3 |
|  | 14 | 107* | C A S S **R L R** A Y D Q Y F G P G T | 2.3 |
|  | 15 | 107* | C A S S **R L R** A Y D Q Y F G P G T | 2.3 |
| 38 EAT | 1 | 108* | C A S S **L G H P** Q D F G P G T | 2.4 |
|  | 2 | 108* | C A S S **L G Q G** Q Y F G P G T | 2.5 |
|  | 3 | 108* | C A S S **L G H P** Q D F G P G T | 2.4 |
|  | 4 | 108* | C A S S **L G H P** Q D F G P G T | 2.4 |
|  | 6 | 108* | C A S S **L G H P** Q D F G P G T | 2.4 |
|  | 7 | 108* | C A S S V S G H Q D F G P G T | 2.4 |
|  | 8 | 108* | C A S S **Q F Q T G R T** Q D F G P G T | 2.4 |
|  | 9 | 108* | C A S S **R L H T** Q Y F G P G T | 2.5 |
|  | 10 | 108* | C A S S **F R A W A R T** Q D F G P G T | 2.4 |

*These sequences resulted from the direct cloning of TRBV28-TRBJ2.5

EAT=epicardial adipose tissue
